# Success and Limitations of Current Force Fields for the Description of RNA–Ligand Complexes

**DOI:** 10.1021/acs.jpcb.5c05262

**Published:** 2025-10-31

**Authors:** Paula Fernández Migens, Israel Serrano-Chacón, Modesto Orozco, Federica Battistini

**Affiliations:** 1 Institute for Research in Biomedicine (IRB Barcelona), Baldiri Reixac 10, Barcelona 08028, Spain; 2 Departament de Bioquímica i Biomedicina, Facultat de Biologia, Universitat de Barcelona, Avgda Diagonal 647, Barcelona 08028, Spain

## Abstract

We present a systematic assessment of the last generation of RNA force fields to reproduce the structures and dynamics of ligand–RNA complexes. Our comprehensive analysis helped not only to define the more reliable force field to represent complex structures but also suggests details that can be improved and provide a critical analysis of the quality of experimental structures in complex systems that are expected to be very flexible and environment dependent.

## Graphical abstract

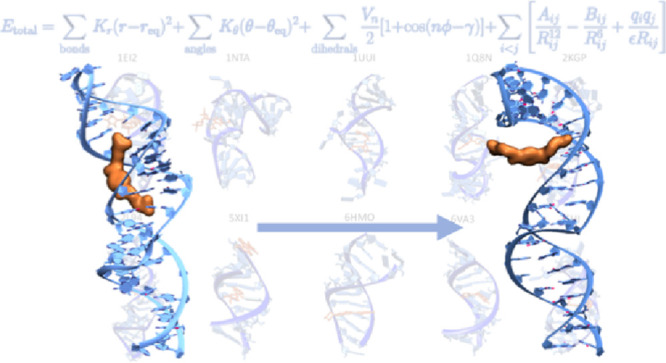

## Introduction

Contrary to DNA, which typically takes the form of a regular double helix, RNA can adopt complex 3D structures, often comparable in complexity to proteins. These structures are crucial for RNA to exert its regulatory functions and have accordingly been refined by evolution with some functionally relevant motives fully conserved. This structural uniqueness has opened the possibility of targeting RNAs with small molecules that, by recognizing specific RNA cavities and grooves, can selectively bind to RNA and mimic the pharmacological strategies traditionally used for protein-targeting drugs. Antibiotics inhibiting protein synthesis were the first RNA binders with therapeutic profile,^1,2^ but in recent years, massive screening campaigns have detected a variety of small molecules interfering with many biological processes and, in some cases, exhibiting promising therapeutic potential.^3–5^

As has been the case for decades with protein ligands, the design of RNA binders would greatly benefit from structure-based approaches. However, this faces the challenge of limited experimental data, which necessitates the use of theoretical methods as well as the lack of well-tuned docking procedures to handle flexible RNA molecules. In this context, atomistic molecular dynamics (MD) appears as the most powerful tool to simultaneously capture structure and dynamics of RNA–drug complexes and uncover the rules that define a good-profile RNA binder.^6^ Current MD protocols benefit from decades of force field (FF) refinement that have transformed MD in a method able to describe a variety of RNA structures, from simple double-helical structures^7^ to more intricate topological tertiary structures.^8^ Interestingly, and quite unique in modeling tools, MD has not only provided information on the structure of RNA, but also on its flexibility^9^ and recognition properties.^10^ Systematic studies have provided detailed estimates of the accuracy expected from FF-derived MD simulations,^11–14^ providing informed users with valuable insight into the predictive power of structural ensembles collected for different RNA motifs. Unfortunately, no such information exists on drug–RNA complexes, and researchers in this field work in a blind manner without any clear expectation of what can be learned from the MD simulation.

In this article, we present a critical analysis of the capabilities and limitations of MD simulations in analyzing drug–RNA complexes. Using representative case studies taken from a curated database of complexes (HARIBOSS),^15^ we tested the state-of-the-art force fields to analyze not only the structural motions of RNA molecules with different topological architectures, but also their interactions and stability when bound to ligands. Overall, we observed that current FFs are generally effective in stabilizing RNA structures without introducing major distortions and tend to improve the energetic interactions with ligands, correcting some local distortions observed in experimentally derived models. The newly refined FFs demonstrate better maintenance of intra-RNA interactions and reduced terminal fraying; however, this occasionally comes at the expense of distorting the RNA experimental model. Regarding RNA–ligand interactions and binding stability, further refinements are still needed to more accurately reproduce experimental observations and achieve consistently stable RNA–ligand complexes. However, our analysis highlights that caution is needed when assuming the experimental structure deposited in the PDB as the definitive conformation without analyzing which aspects of the structure are supported by direct experimental data and which ones are derived from a refinement process performed under a limited number of experimental restraints.

## Methods

### Structure Selection and Preprocessing

RNA–ligand structures were extracted from HARIBOSS, a curated database of RNA complexes with drug-like ligands determined by X-ray crystallography, NMR spectroscopy, and cryo-electron microscopy. To ensure a diverse data set, structures were selected to capture a range of RNA architectures, binding mode, and chemical diversity of the ligands. The PDB ID code of the structures selected are 1EI2,^16^ 1NTA,^17^ 1Q8N,^18^ 1UUI,^19^ 2KGP,^20^ 2L94,^21^ 5XI1,^22^ 6HMO,^23^ 6VA3,^24^ and 7FHI.^25^ The structures selected together with the experimental method, RNA sequence, ligand details, and RNA 2*D*/3D structure are summarized in Figure 1 and Supplementary Table S1. For consistency across structures and to study canonical base pairing, pseudouridines in the 6HMO RNA structure were replaced with standard uridines; this mutation did not directly affect the binding site being far away from the RNA–ligand interaction region.

**Figure 1:**
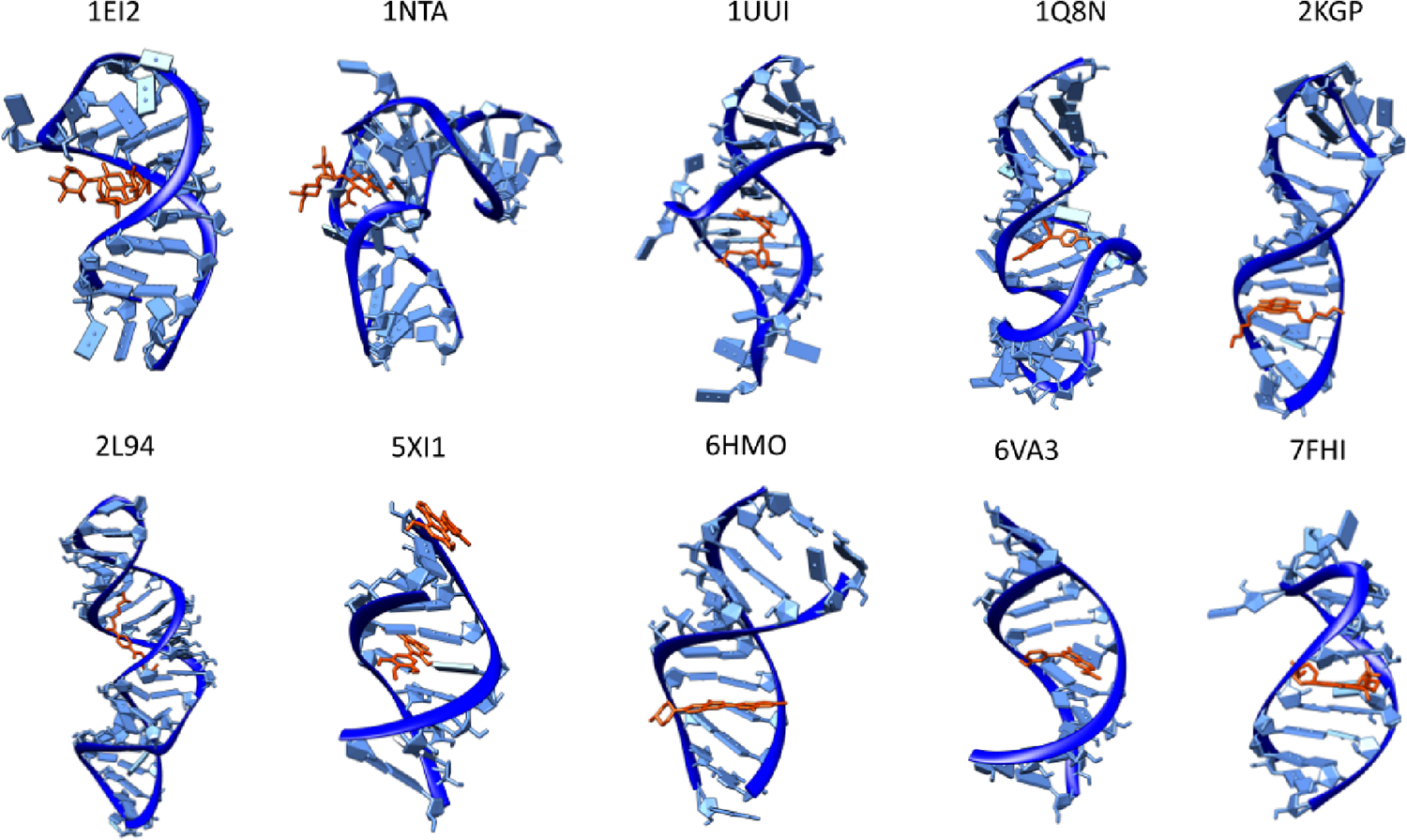
Representation of the selected structures: 1EI2, 1NTA, 1Q8N, 1UUI, 2KGP, 2L94, 5XI1, 6HMO, 6VA3, and 7FHI. The RNA backbone and bases are colored in blue respectively while the ligand in licorice (in orange) (see Supplementary Table S1 for more details on ligand, sequence, and 2D structure).

### Molecular Dynamic Simulation Set up

RNA–ligand systems were simulated for 1 μs using Amber 20 ^26^ or GROMACS 2023 ^27^ depending on the FF, Amber 20 for OL3,^28^ De Shaw,^29^ and OL3cp ghbfix21 ^30,31^ force fields, while DES-AMBER^32^ was simulated using GROMACS 2023. Ligand parameters were parametrized using ACpype^33^ and Gaussian 16 ^34^ and GAFF2 force field^35^ with RESP2 charges,^36^ which permit direct comparison with the different force fields without noise arising from different parametrization protocols for the ligand. Protonation state of the ligands was defined using Marvin.^37^ All systems run with Amber 20 were solvated in an octahedral OPC water box built with a 15 Å padding parameter, neutralized with K^+^ ions, and set to a near-physiological 0.15 M concentration of K^+^ and Cl^–^ ions. The systems underwent standard optimization procedures, including energy minimization, thermalization, and a final 10 ns re-equilibration, before performing 1 μs unrestrained MD simulations under NPT conditions (1 atm, 298 K).

gHBfix21 was applied only in the OL3cp simulations. Sequence-specific gHBfix inputs were generated and activated during production via PLUMED^38^ coupling in Amber. No gHBfix terms were used in the OL3 or Shaw runs. For DES-AMBER, run with GROMACS 2023, systems were solvated in a dodecahedral box of TIP4P-D water (15 Å padding), and ionized to ∼150 mM KCl (K^+^/Cl^–^ carry ± 0.9e under DES charge scaling). Because the total box charge is not always divisible by ±0.9e, residual net charge was minimized (always <0.5e) using gromologist^39^ and handled by PME’s uniform neutralizing background. Energy minimization and system preparation were performed through the same automated gromologist workflow. Production MD (1 μs, NPT) was launched directly from minimized structures (velocities generated at 300 K; no separate preproduction equilibration). Simulations used the velocity-rescale thermostat (τ = 0.1 ps, 300 K) and Berendsen isotropic barostat (τ = 2.0 ps, 1 bar). PME electrostatics employed a 1.0 nm real-space cutoff; van der Waals interactions used a 1.0 nm cutoff (dispersion correction off).

### RMSD and LoRMSD Calculations

RMSD calculations were performed using VMD. All RMSD values were computed with respect to the experimental structure, considering only heavy atoms. For systems with multiple NMR conformers, the one with the lowest RMSD was selected as the representative structure. Additionally, the RMSD was calculated with respect to the average structure of the MD trajectory. Ligand mobility relative to RNA was quantified using ligand-only RMSD (LoRMSD): trajectories were aligned to the experimental RNA backbone, and the heavy-atom RMSD of ligand residues was measured.^40^

### RMSF

All RMSF values were calculated directly from the molecular dynamics trajectories as the deviation of each atom from its time-averaged position across the trajectory (in Å), using the MDAnalysis package.^41,42^ Atoms corresponding to the ligand were excluded from the analysis.

### RNA Parameters

Helical parameters: major groove width, twist, and puckering (% north) were computed with pytraj (nastruct).^43^

### Contact Map Analysis

Contact maps were generated using the Contact Map Explorer Python package.^44^ MD trajectories for all force fields were processed to compute residue–residue contacts.

A residue–residue contact is defined when the minimum distance between any pair of heavy atoms from two noncontiguous residues is less than 4.5 Å. An average contact map was obtained by calculating the fraction of frames in which a contact was observed across the entire trajectory. For NMR-derived experimental structures, all available NMR models were treated as a single trajectory to capture the full range of conformations, consistent with NMR restraints. An average contact map was then computed to reflect the overall residue–residue interaction patterns across all models. For each structure, the contact map corresponding to the initial experimental PDB model was subtracted from the average contact map obtained from MD simulations for each FF. The resulting delta map reflects the change in contact frequency for each residue pair, with positive values indicating contacts gained during the simulation relative to the starting structure and negative values indicating contacts lost.

### Counts of Contacts and Contact-Occupancy Variation (σ)

The initial ligand-RNA contacts are defined from the PDB starting structure. For every heavy-atom contact, we calculated its occupancy (1 = present, 0 = absent) for each frame of the trajectory and used the average of this series to generate the average contact maps described above. A native contact was considered lost if its occupancy was <0.10; a contact was gained if a ligand–RNA pair absent in the starting structure reached an average occupancy ≥0.10. The corresponding standard deviation was estimated from the variability of contact occupancies along the trajectories.

The contact-occupancy variation (σ) is defined as the standard deviation of the occupancy time series, which captures how much the occupancy fluctuates over time. Thus, σ provides a direct measure of contact stability without any threshold: σ = 0 indicates a perfectly stable contact (always present or always absent), while σ increases with variability, reaching a maximum of 0.5 when a contact is present in roughly half of the frames and absent in the other half.

### Data Availability

All MD simulations conducted in this study have been uploaded to MDposit^45^ in a FAIR format,^46^ an open-access platform designed to provide web-based access to atomistic MD simulations. Additionally, through the workflow available on the MDposit Web site, a wide range of analyses were performed on the simulations, including the calculation of molecular mechanics (MM) energies. The energy analysis on MDposit is conducted using CMIP.^47^

## Visualization and Figure Preparation

All molecular-dynamics trajectories were inspected, and every image used in this work was produced with Visual Molecular Dynamics (VMD) v1.9 ^48,49^ and UCSF Chimera v1.17.^50^

## Results and Discussion

We studied 10 RNA–small molecule structures selected from the HARIBOSS curated database, which compiles entries from the PDB for structural-based drug design. The selected systems encompass a diverse range of RNA topologies, including double helices, hairpins, and chemically distinct binders (both hydrophobic and hydrophilic), with various binding modes, such as intercalation and groove binding. In details we defined groove binders as the complexes with ligands that recognize and bind within the major or minor grooves of RNA, while intercalators are planar aromatic molecules that insert between adjacent base pairs of nucleic acids (Figure 1 and Supplementary Table S1).

For this study, we selected four FFs to test, choosing among the most commonly used or more recently refined. Particularly, OL3, an AMBER force field, includes specific updates such as the parmbsc0 α/γ torsion correction and a refit of the glycosidic χ torsion (χ-OL3) to improve backbone and base orientations. Its newly refined version, OL3cp ghbfix21, referred to in this article as OL3cp, builds on OL3 by incorporating the case phosphate (CP) nonbonded parameters, which soften phosphate-base contacts via revised charges and matched dihedrals, and by adding the gHBfix21 correction, a data-driven modification that slightly enhances canonical base-pair hydrogen bonding. Shaw’s FF (also referred to as DESRES in the literature and as Shaw in this article) represents a full reparameterization including extensive refitting of torsional angles (χ, γ, ε/ζ, β, and sugar-pucker), replacement of standard phosphate van der Waals terms with CP phosphate parameters, implementation of a large NBfix table to fine-tune base-stacking and base-phosphate contacts, and adjustments of selected nucleobase charges and LJ radii to better balance hydrogen bonding and π-stacking. DES-AMBER (here referred also as DES) offers a charge-damped approach, in which all partial charges are uniformly scaled to 0.9, assuming that this would help to correct errors derived from the lack of electronic polarization in Lifson’s like FFs.^51,52^ In addition, the χ and ζ torsions are refitted using MP2-level quantum data, and a minimal NBfix patch is applied to selectively adjust Lennard-Jones interactions between phosphate oxygens and aromatic carbons with water, helping to preserve base stacking and solvation behavior under the softened electrostatics.

For each complex, a 1 μs simulation in explicit solvent was run (details in Methods), starting from the experimental PDB structure (see Methods). Overall, throughout the simulations, the RNA exhibited stable dynamics (Table 1 and Supplementary Figure S1) deviating from the experimental structure by an average RMSD around 4.53 ± 0.97 Å (OL3), 4.71 ± 0.99 Å (Shaw), 3.78 ± 0.92 Å (OL3cp), and 4.5 ± 1.1 Å (DES- AMBER). The FFs also demonstrated RNA stability throughout the simulations when compared to the average structure, with low RMSD values of 1.92 ± 0.60 Å for OL3, 1.91 ± 0.32 Å for the Shaw FF, 1.90 ± 0.44 Å for OL3cp, and 2.25 ± 0.53 Å for DES-AMBER (Table 1 and Supplementary Figure S2). Regarding the RMSD examining the values for each complex, OL3cp generally appears to be the best-performing FF, but deviations obtained from the other FFs are in general moderate (Supplementary Figures S1–SS2).

**Table 1:** RMSD (Å) of RNA Heavy Atoms Using the OL3, Shaw, OL3cp, and DES-AMBER Force Fields^a^

PDB ID	OL3 (Å)	Shaw (Å)	OL3cp (Å)	DES (Å)
*1EI2*	5.13 ± 0.43	5.12 ± 0.34	3.51 ± 0.35	3.50 ± 0.39
	2.41 ± 0.67	1.57 ± 0.27	1.78 ± 0.37	1.79 ± 0.52
*1NTA*	4.70 ± 0.39	4.62 ± 0.39	3.16 ± 0.63	3.85 ± 0.76
	1.62 ± 0.42	2.03 ± 0.58	1.99 ± 0.68	2.03 ± 0.58
*1Q8N*	5.93 ± 0.37	6.46 ± 0.47	5.31 ± 0.58	6.99 ± 0.65
	1.80 ± 0.48	1.81 ± 0.51	1.81 ± 0.51	2.31 ± 0.54
*1UUI*	3.92 ± 0.47	5.61 ± 0.63	4.03 ± 0.63	4.28 ± 0.54
	1.08 ± 0.28	1.75 ± 0.51	0.97 ± 0.28	1.80 ± 0.56
*2KGP*	5.11 ± 0.74	4.98 ± 0.77	5.37 ± 0.73	5.09 ± 0.69
	1.77 ± 0.46	2.02 ± 0.71	2.08 ± 0.40	2.40 ± 0.46
*2L94*	3.62 ± 0.70	3.25 ± 0.67	3.92 ± 0.63	5.7 ± 1.9
	2.47 ± 0.61	2.36 ± 0.51	1.83 ± 0.47	2.04 ± 0.53
*5XI1*	2.50 ± 0.44	3.13 ± 0.79	2.47 ± 0.45	4.53 ± 1.1
	2.55 ± 0.67	2.49 ± 0.66	2.51 ± 0.63	3.5 ± 1.5
*6HMO*	5.15 ± 0.60	4.58 ± 0.24	3.31 ± 0.56	3.63 ± 0.69
	1.39 ± 0.47	1.58 ± 0.55	1.70 ± 0.37	1.70 ± 0.70
*6VA3*	4.65 ± 0.24	4.69 ± 0.33	4.88 ± 0.29	4.85 ± 0.38
	2.83 ± 0.54	1.93 ± 0.43	2.47 ± 0.52	2.66 ± 0.52
*7FHI*	4.56 ± 0.18	4.65 ± 0.28	3.36 ± 0.51	5.33 ± 0.52
	1.29 ± 0.30	1.58 ± 0.44	1.63 ± 0.35	2.29 ± 0.45
*Average*	4.53 ± 0.97	4.71 ± 0.99	3.93 ± 0.97	4.7 ± 1.1
	1.92 ± 0.6	1.91 ± 0.32	1.90 ± 0.44	2.25 ± 0.53

a Each cell shows RMSD relative to the experimental structure (top) and to the average structure from the trajectory (bottom).

When examining the magnitude of atomic motions along the trajectories relative to their mean positions (RMSF, see Methods, Table 2, and Supplementary Figure S3), we observed highly similar fluctuation profiles across the different force fields (FFs). Overall, the simulations were stable with larger deviations restricted to the terminal residues. The overall average RMSF values indicate that OL3, Shaw, and OL3cp produce comparable levels of structural stability (1.72–1.81 Å), whereas DES consistently yields higher fluctuations (2.13 Å on average), reflecting a tendency toward greater flexibility. Among the individual cases, OL3cp generally produced the lowest fluctuations relative to the experimental references, while DES showed the largest deviations, particularly for 2L94, where the average RMSF exceeded 3.5 Å.

**Table 2:** RMSF (Å) of RNA Using OL3, Shaw, OL3cp, and DES-AMBER Force Fields for Each Experimental Structure (PDB ID) and Overall Average (Bottom)^a^

structure	OL3 (Å)	Shaw (Å)	OL3cp (Å)	DES (Å)
1EI2	2.08 ± 1.50	1.45 ± 0.78	1.58 ± 0.97	1.72 ± 0.87
1NTA	1.53 ± 0.78	1.91 ± 1.03	1.91 ± 1.03	1.95 ± 0.90
1Q8N	2.90 ± 1.01	1.91 ± 0.87	1.99 ± 0.75	2.15 ± 1.08
1UUI	1.45 ± 0.70	1.95 ± 0.94	1.75 ± 1.25	2.04 ± 1.41
2KGP	2.24 ± 1.29	2.12 ± 1.28	1.72 ± 0.87	1.93 ± 0.96
2L94	2.53 ± 0.88	2.41 ± 1.00	2.49 ± 0.83	3.67 ± 1.14
5XI1	1.29 ± 0.55	1.32 ± 0.53	1.00 ± 0.35	1.79 ± 0.76
6HMO	1.39 ± 0.65	1.40 ± 0.64	2.18 ± 1.42	2.30 ± 1.56
6VA3	1.41 ± 0.61	1.41 ± 0.61	1.52 ± 1.01	1.70 ± 0.81
7FHI	1.32 ± 0.59	1.35 ± 0.58	1.60 ± 0.58	2.09 ± 1.14
**Mean**	1.81 ± 0.58	1.72 ± 0.38	1.77 ± 0.41	2.13 ± 0.57

a Profiles are represented in Supplementary Figure S3.

Hydrogen bond analysis (Table S2 and Figure S4) revealed that canonical base pairing was generally well maintained across the simulations. A·U pairs were consistently more labile than G·C pairs, as expected, with average hydrogen bond losses for A·U pairs ranging from ∼5 to 10% depending on the FF. G·C pairs were more stable, typically showing losses below 2% for 1H and 2H, although the third hydrogen bond displayed higher variability (up to ∼5–9% in OL3). Importantly, OL3cp displayed a clear improvement over OL3 in stabilizing G·C pairs, where hydrogen-bond losses were reduced and geometries remained closer to the experimental reference. DES-AMBER, on the other hand, showed the most consistent ability to maintain canonical hydrogen bonds across different systems, often outperforming other FFs in terms of base-pairing stability, even though this came at the cost of distortions in helical shape (see below). Overall, no reproducible sequence-dependent effects were identified, with the only minor case being slightly higher instability of A·U pairs. Instead, the main differences are better explained by the presence or absence of ligands, as discussed in the following section.

Analysis of the flickering of contacts RNA–RNA (Supplementary Figure S5) revealed no substantial differences among the FFs. All displayed similar levels of transient contact formation and disruption, with Shaw showing somewhat greater variability.

Regarding helical parameters, we generally observed maintenance of groove dimensions (major groove width, Supplementary Figure S6), except for DES FF. While DES preserved hydrogen-bonding patterns, it did so at the expense of helical geometry, leading to widening of the major groove and progressive deviation from the experimental RNA conformation. At the level of interbase geometry (twist) and sugar puckering (%North conformation), all FFs maintained the experimental features with only minor variations (Supplementary Figures S7–S8).

Overall, the different FFs showed good performance, although some limitations, detailed below, may have a non-negligible impact on the predicted RNA–drug binding modes.

In detail, across the simulations, major changes in the RNA structure were detected primarily at the terminal regions when compared to the experimental structure (see Methods for details), which becomes evident when looking at the maintenance of the hydrogen bond pattern throughout the simulations (see Suppl. Figure S4). Some structures show systematic corruption of RNA intramolecular H-bonds (for example, 6HMO), while others, like 5XI1, remain fully stable RNA secondary structures. In fact, differences related to the structure considered are larger than those arising from the FF (see Suppl. Figure S4). It is worth noting how even small distortions in the hydrogen bond pattern found in one FF are detected also in the others (see, for example 2L94). This cross-validation between different FFs reinforces the robustness of the MD simulations and suggests that some experimental structures may need to be re-examined or that the experimental structure could be influenced by the conditions under which it was obtained; however, it is also possible that all force fields introduce a systematic bias, a possibility that should be considered when interpreting the results.

Interesting effects are detected when the analysis is focused on specific structures. For example, many simulations show opening at central steps, as commented above, mainly A·U steps (ex. 1NTA, 1UUI, 2L94, or 7FHI), especially for Shaw and OL3/OL3cp FFs. These openings primarily coincide in the experimental structures with unstable hydrogen bonds, either due to the distorted RNA secondary structure or as a result of competition with the ligands. In the case of 1NTA, the open pair (19U·27A) is situated in asymmetric internal loops, adjacent to an unpaired base, with less stable noncanonical hydrogen bonds, as supported by the experimental data (see Suppl. Figures S4 and S9). In the other cases, even if there is not clear evidence from the experimental structure of these openings or weak bonds, they are present in the distorted internal loop or intercalation site, with ligand interactions (2L94 and 7FHI), where *ad hoc* restraints were applied in the resolution (1UUI) (see Suppl. Figures S4 and S10).

As mentioned previously, one case especially remarkable is 6HMO, a splice-site duplex, where some terminal hydrogen bonds present in the NMR structure were lost in the different FF simulations, while new ones formed (Suppl. Figure S4). These changes are generally attributed to a rearrangement in the RNA along the simulations, particularly in the terminal region. These rearrangements at the terminal end allow new hydrogen bonds to form (see Suppl. Figure S11) and enhance stacking with a residue that was initially fully exposed to the solvent. Furthermore, this terminal region consists of an A·U pair; a C·U mismatch, experimentally shown not to form stable base pairs; and an A·U pair, creating an inherently flexible arrangement prone to structural fluctuations. It is important to note that these rearrangements are not directly linked to ligand movement. However, the ligand moves throughout the simulation and behaves differently depending on the FF used (as discussed below). The RNA-level deviations suggest moderate instability in the terminal structure along with the simulations. However, the uniform behavior across FFs appears to be influenced by the lack of strong experimental base-pairing restraints at specific sites in the NMR structure.

Less dramatic, but remarkable, PDB entries 1EI2 and 2KGP, which involve the same RNA moiety, show high variability at the terminal part and instability in the hydrogen bonds (Figure 2). During the simulations, the base pairing in this terminal region varies depending on the FF used. For 1EI2, the MD simulation using OL3 shows the terminal part unpaired throughout the trajectory, while the structure evolves to compensate for stable stacking between the terminal bases, which might signal some unbalance in H-bond/stacking interactions in this FF. In contrast, the other FFs show lower values of RMSD and more stability in the H-bond pattern. Shaw and OL3cp recover a canonical H-bond pattern (see Suppl. Figure S4), while DES-AMBER shows initial pairing but exhibits some loss of stability, with extended fraying occurring midway through the simulation before recovering later on. For 2KGP, again, OL3 showed low stability at the terminal regions and were more affected by fraying, with disruption until the third base pair (a G·C one), which goes against the experimental evidence that determined this pairing to be stable upon the ligand binding. In contrast, the other FFs maintained more stable base pairing to varying degrees with reversible fraying occurring during the simulation and involving RNA regions distant from the ligand.

**Figure 2:**
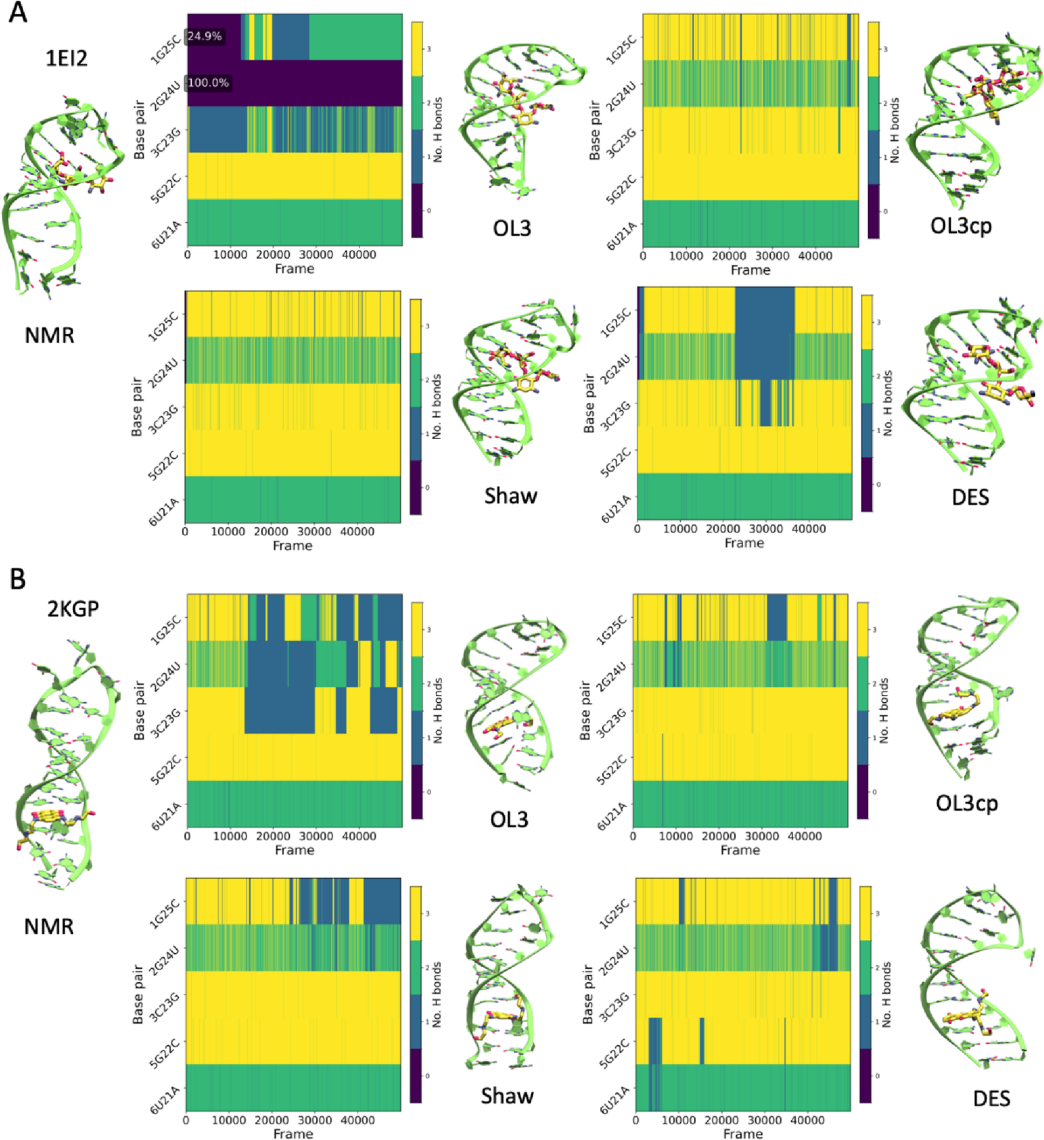
Time evolution of canonical and noncanonical base-pair H-bonding in two RNA–ligand complexes. (A) 1EI2 and (B) 2KGP. Top: Heatmaps show the number of hydrogen bonds, both canonical and noncanonical, of terminal base pairs over time for four force fields (OL3, Shaw, OL3cp, and DES-AMBER). Each row corresponds to a base pair from the NMR starting structure; columns represent the simulation frames. Color scale: 0 (purple), 1 (blue), 2 (green), and 3 (yellow). Bottom: Representative snapshots were from the end of each simulation.

Finally, base pairing is fully maintained across all FFs in structures 5XI1, 6VA3, and 1Q8N, which include two hairpins and one double-helical RNA with intercalators. However, the maximization of ligand intercalation can, in some cases, affect the RNA conformation and its A-form shape, as will be explained in more detail in the following section.

### RNA–Ligand Interactions

The choice of FF affects the ligand-binding stability along the simulations. As previously mentioned, our structure selection included a diverse set of ligands featuring various chemical groups and binding modes (see Supplementary Table S1). This selection was designed to include both groove binders and intercalators, and for clarity, we analyzed these two categories separately, given the different types of RNA–ligand interactions involved. The refinement of the FFs, particularly in their treatment of electrostatics and stacking interactions, clearly influenced RNA-binding mode, as shown in LoRMSD (see Methods for details) values in Suppl. Figure S12. Adjustments such as improved charge distribution on phosphate groups and enhanced base stacking were reflected in shifts in binding poses. These effects are especially relevant for more dynamic ligands or weakly constrained binding pockets.

The most pronounced variation in contacts was observed for groove binders, as visible in Figure 3, where the variation (σ) (see Methods) of the frame-by-frame occupancy of each ligand–RNA heavy-atom contact is depicted. DES-AMBER and Shaw violins broaden out to σ ≈ 0.45 (σ = sd. of contact occupancy) while those for OL3 and OL3cp remain centered below σ ≈ 0.15, reflecting more persistent ligand–RNA contacts in the latter two FFs, while DES-AMBER and Shaw FFs showed the poor ability to maintain ligand-RNA interactions, instead favoring compaction of the RNA and intra-RNA interactions over ligand retention. Regarding intercalators, the initial poses and contact patterns were largely maintained by almost all FFs. DES-AMBER showed the greatest variation in this case.

**Figure 3:**
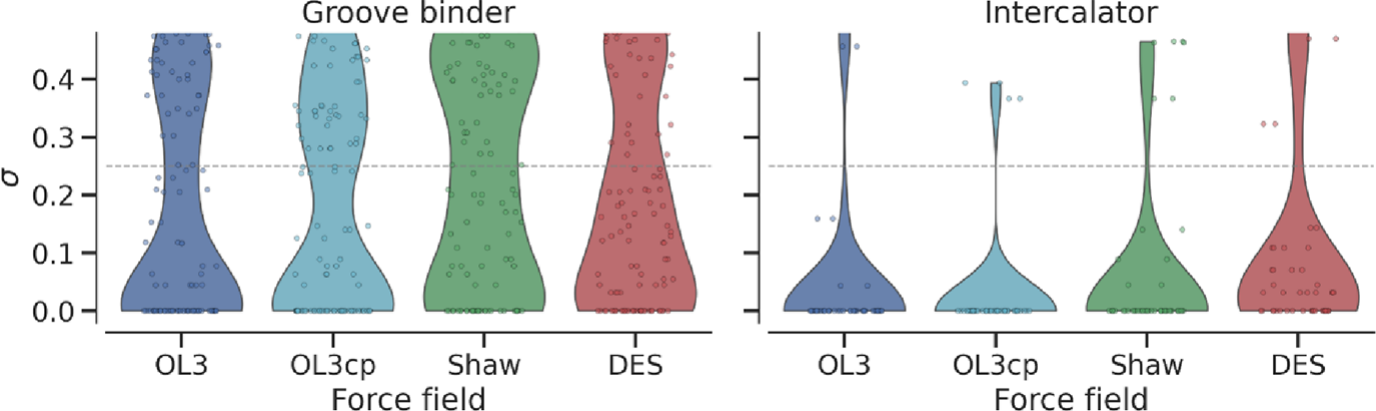
Ligand-RNA contact stability across the trajectory: σ is the standard deviation of the frame-by-frame occupancy of each ligand–RNA heavy-atom contact: a value of 0 means the contact is maintained throughout the trajectory, whereas values that rise toward 0.5 reflect increasingly “flickery” contacts that break and reform repeatedly. The violins display the σ distributions for groove binders (left) and intercalators (right) in the four FFs. Colors correspond to the four FFs: OL3 (dark blue), OL3cp (light blue), Shaw (green), and DES-AMBER (red).

As the behavior of groove binders and intercalators is very different, in the following, we will describe separately the behavior of these two families of complexes.

### RNA–Ligand: Groove Binders

The groove binders (PDB IDs 1EI2, 1NTA, 1UUI, 2L94, and 6HMO; see Supplementary Table S1) remained mostly bound throughout all simulations. However, the pattern of interactions diverges in several cases from the experimental ones, as seen in the number of contacts gained/lost relative to the starting structure (see Methods and Figure 4). As noted earlier, OL3cp generally preserved experimental contacts better than any other FF, but there is notable variability depending on the complex.

**Figure 4:**
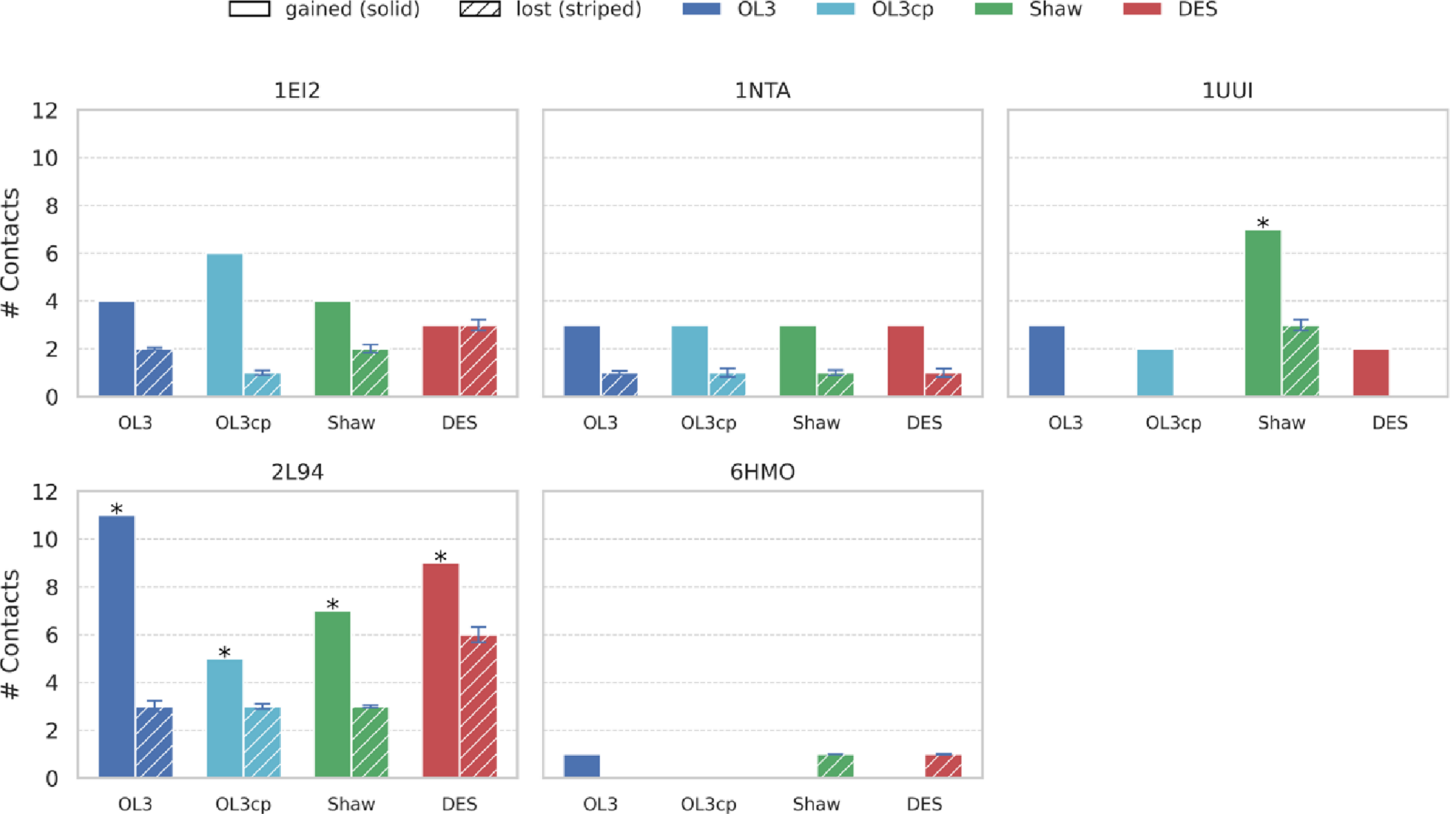
Changes in ligand–RNA contacts under different FFs for groove binders. Histograms showing the number of starting ligand contacts gained (full color) or lost (dashed color) (present in the experimental structure but broken in >10% of simulation frames). Bars: OL3 (dark blue), OL3cp (light blue), Shaw (green), DES-AMBER (red); PDB IDs above panels. Asterisks mark trajectories in which the ligand became unbound. For details on the contacts gained and lost, see Suppl. Figures S13–S15.

In the 6HMO structure, pronounced differences in ligand-RNA interactions emerge between FFs, as the ligand exhibits a notable change in binding mode during the simulations. The ligand is a small molecule (named SMN-C5, see Supplementary Table S1) that interacts with the RNA duplex primarily through its positively charged piperazine group, anchoring the molecule to RNA’s C9 phosphate and its central planar aromatic ring forming a hydrogen bond with an unpaired adenine. The key interaction between the RNA phosphate and the charged piperazine group is maintained across all simulations, but with more intercalation compared to the NMR structure. We encountered, however, different behaviors regarding the interaction with the bulged-A. Examining the raw experimental data, we noted that the ligand interacts with the unpaired A, but during the structure determination, a hydrogen bond between the ligand and A14 was forced in the refinement model structure. This hydrogen bond was not respected by any of the FFs. Nevertheless, both the Shaw and DES-AMBER force fields maintain the interaction without introducing additional distance violations, although the bulged-A is displaced toward the groove. Except for the enforced hydrogen bond, the remaining NOE restraints are satisfied. Simulations using OL3 and OL3cp show a deeper intercalation of the ligand that causes the movement of the A-bulged groove to the opposite groove (A14 in Figure 5). Re-examining the NMR restraints available for 6HMO in the PDB, we found that the new intercalated binding mode observed in OL3 and OL3cp simulations is mainly compatible with the experimental NMR data, just losing the interactions with the A-bulge.

**Figure 5:**
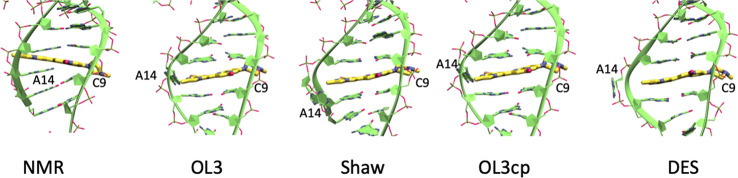
Force field-dependent ligand rearrangement in 6HMO. From left to right are representative snapshots of NMR, OL3, Shaw, OL3cp, and DES-AMBER (DES).

Major variations in the ligand binding using different FFs have been detected for PDB ID 2L94, a frameshift hairpin, showing the greatest variation in contacts gained and lost throughout the simulation (see Figure 6). The ligand is a small, rigid, tetracationic bis-guanidine compound that binds in the major groove of HIV-1 FS RNA, forming hydrogen bonds with phosphate oxygens on opposite sides and extensive van der Waals contacts. This interaction stabilizes the RNA, spans 9 base pairs, and alters the conformation of a conserved GGA bulge. The ligand is anchored to the groove through two interactions, hot spots, between adenine 27 and uracil 10, and the two guanidinium groups of the ligand. All these interactions are directly supported by strong NMR signals. Overall, the RNA maintains a hairpin shape along the simulation (Figure 6), with only a central weak pairing (Suppl. Figure S10). Regarding the ligand binding, however, we can see a very unstable binding (see LoRMSD and delta contact maps in Figure 6). OL3cp and Shaw are able to rearrange the complex, still maintaining one of the two hot spots clearly detected in the experimental structure. On the contrary, OL3 and DES-AMBER show a more drastic deviation, as indicated by the LoRMSD profile (see Figure 6). For this structure, both of these FFs lead to a noticeable change in contacts compared to the experimental reference. The DES-AMBER FF loses all RNA–ligand contacts at the start of the simulation, but recovers one of the original interactions by the end of it (Figure 6); OL3 simulations show a dramatic detachment of the ligand from experimentally characterized binding sites. This change of the ligand binding is not supported by experimental evidence; however, interestingly, none of the FFs are able to maintain the experimental binding pose and the interactions detected by NMR.

**Figure 6:**
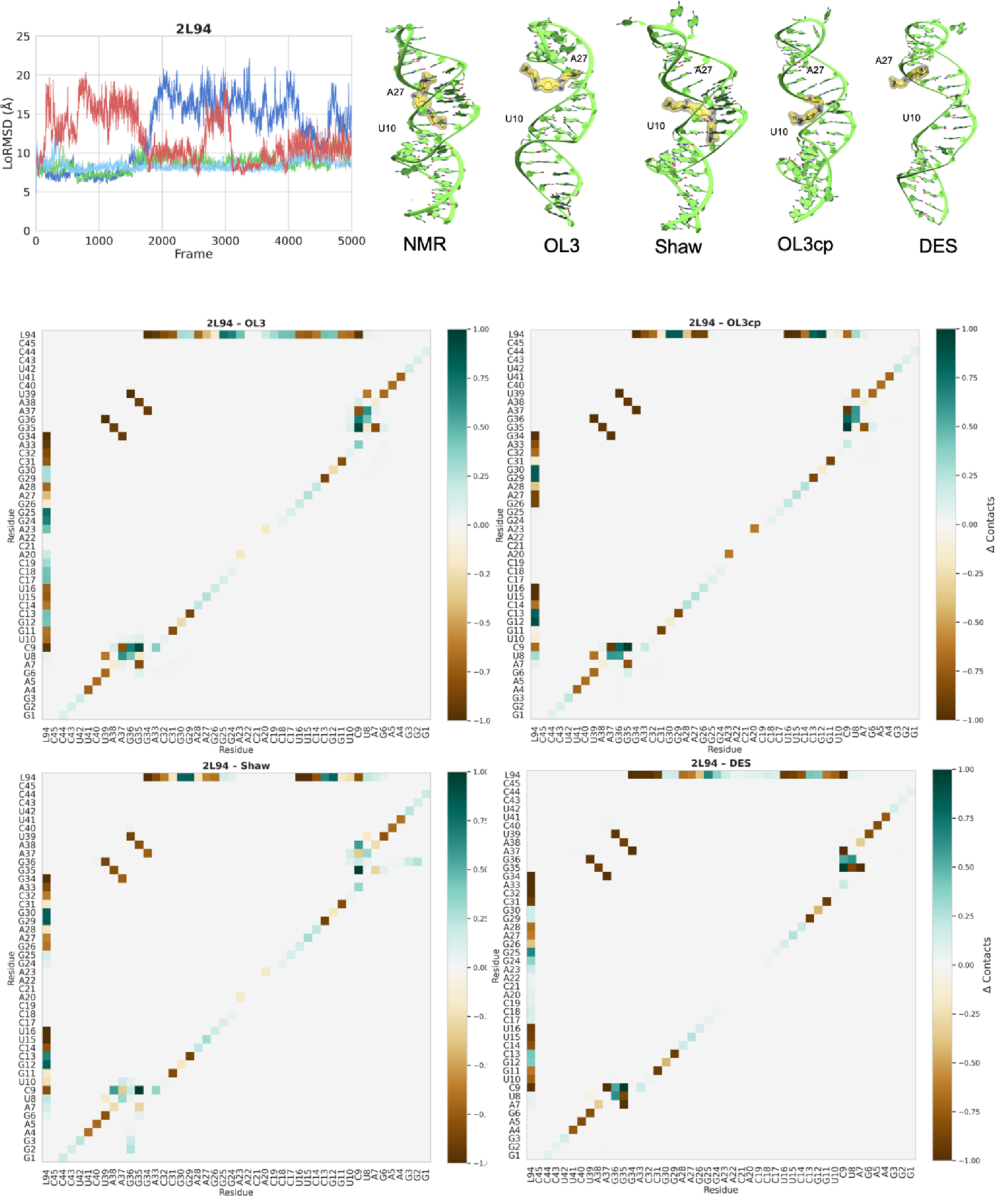
Analysis of ligand–RNA binding for the 2L94 structure using OL3 and DES-AMBER FFs. Top panels, from left to right, LoRMSD profiles for all FFs along the simulation (in Å), Delta contact maps along the simulation of RNA–RNA and RNA–ligand contacts using OL3, Shaw, OL3cp, and DES FFs respectively. Positive Δ (green) denotes contacts gained; negative Δ (brown) denotes contacts lost. Bottom panel, from left to right, structures of the complex with the ligand in yellow and the two hot spot residues A27 and U10 marked: NMR structure and final structure using OL3, Shaw, OL3cp, and DES FFs, respectively. To illustrate the different poses of the ligand using the various FFs, the images were generated by aligning the structures based on the RNA.

Another example of ligand binding loss along the MD simulation is observed in PDB ID 1UUI. In complex, the ligand is a small bis-guanidine compound that targets HIV-1 TAR RNA. The complex is stabilized by two electrostatic hot spots in the RNA’s major groove that involve positively charged guanidinium groups stacking underneath and above U23 (here renumbered U7). As reflected in the LoRMSD values (see Suppl. Figure S12), the only force field that fails to maintain the RNA structure and ligand binding is Shaw (see Figure 7 and Supplementary Figure S13). With this FF, the RNA tends to compact and prioritize intra-RNA interactions, which negatively impact ligand binding, in clear contradiction with direct experimental evidence. Although in the determination of the structure Watson and Crick (WC) hydrogen bonds restraints have been applied without allowing the sampling of different conformations, the binding site and interactions of the ligand with the RNA (hot spots, U7, A6, G12 and A11, see Figure 7) are clearly assigned and three of them are lost in the Shaw MD simulation. Using the other FFs, we could not detect major variations, and the structures along the simulations agreed with the experimental one.

**Figure 7:**
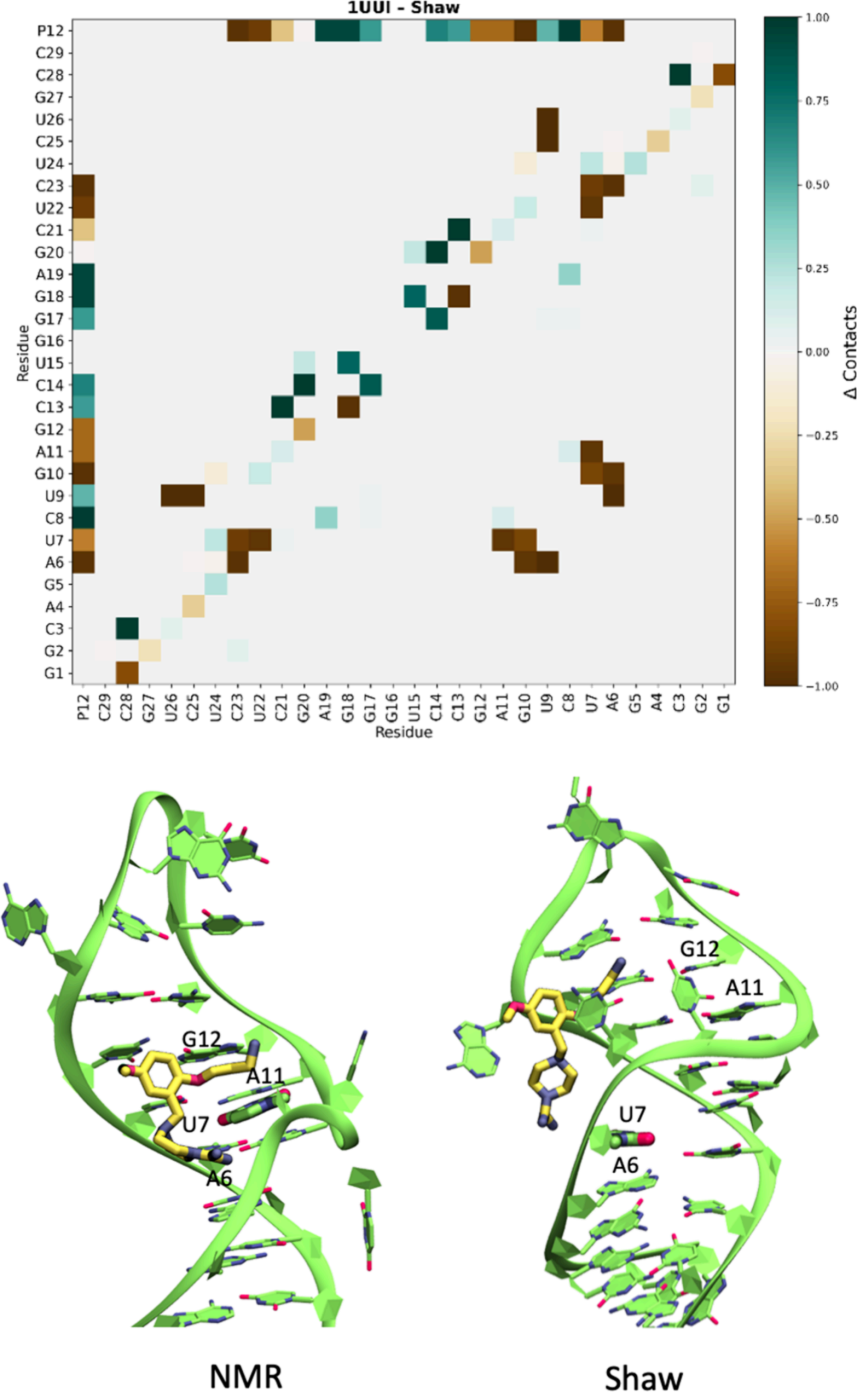
Analysis of the ligand-RNA binding for the 1UUI structure using Shaw FF. In the top panel, the delta contact map shows the variation in density between the start and along the simulation of the contacts RNA–RNA and RNA–ligand. On the bottom panel, starting structure (left) and final structure (right), showing the closing of the groove and the partial unbinding of the ligand.

Less pronounced rearrangements and variations have been detected for PDB ID 1EI2, where a positively charged aminoglycoside antibiotic (neomycin B) binds in the RNA major groove. The complex is stabilized by generic electrostatic interactions supplemented by extensive van der Waals contacts and specific hydrogen bonds with RNA bases and phosphates. As described in the previous section, OL3 does not form the hydrogen bonds in the terminal region of the 1EI2 structure and does not cause major changes in ligand binding (see Figure 2A). Also using DES, we detected, as explained above, a deep fraying along the simulations but not a major variation in the ligand binding. Shaw FF deviates also from experimental structure, but in this case, the increase is due to stabilization of base pairing, opposite to OL3, and enhanced interactions with the ligand. OL3cp increases the contacts between the ligand and the phosphate group, eventually extending those contacts to residues 3 and 4 during the simulation compared to the starting conformation (see Suppl. Figures S13–S15). These interactions are not supported by experimental data and could be due to the experimental conditions and high variability of the ligand poses or to the maximization of the interaction with the phosphate groups. Finally, PDB ID 1NTA corresponds to an RNA aptamer that binds streptomycin encapsulated within the RNA aptamer’s cylindrical pocket (see Suppl. Figure S9). In the experimental X-ray crystal structure, it forms direct hydrogen bonds with RNA base edges, 2’–OH groups, and a backbone phosphate. Along the simulations, the ligand remains in the binding pocket regardless of the force field used, without any significant variation. Only a local rearrangement is observed, which appears to improve both van der Waals and electrostatic interactions between the ligand and RNA (see Supplementary contact and delta contact maps Figures S14–S16). Notably, OL3cp is the FF that shows the lowest RMSD and LoRMSD value.

Overall, regarding groove-binders, OL3cp appears to be the most reliable FF, but even for this FF, non-negligible discrepancies from the experiments are detected. In contrast, OL3 and Shaw show more variations and a greater loss of native RNA–ligand contacts and for OL3 mainly due to enhanced intercalation.

### RNA–Ligand: Intercalators

The intercalator complexes (PDB IDs 1Q8N, 2KGP, 5XI1, 6VA3, and 7FHI) (see Supplementary Table S1) remain quite stable during the trajectories, with ligands remaining close to their experimental binding mode, reflecting probably the strength of stacking interactions. However, even in these cases, the FFs exhibit distinct trends, strengths, and weaknesses that can influence the quality and stability of ligand binding (Figure 8).

**Figure 8:**
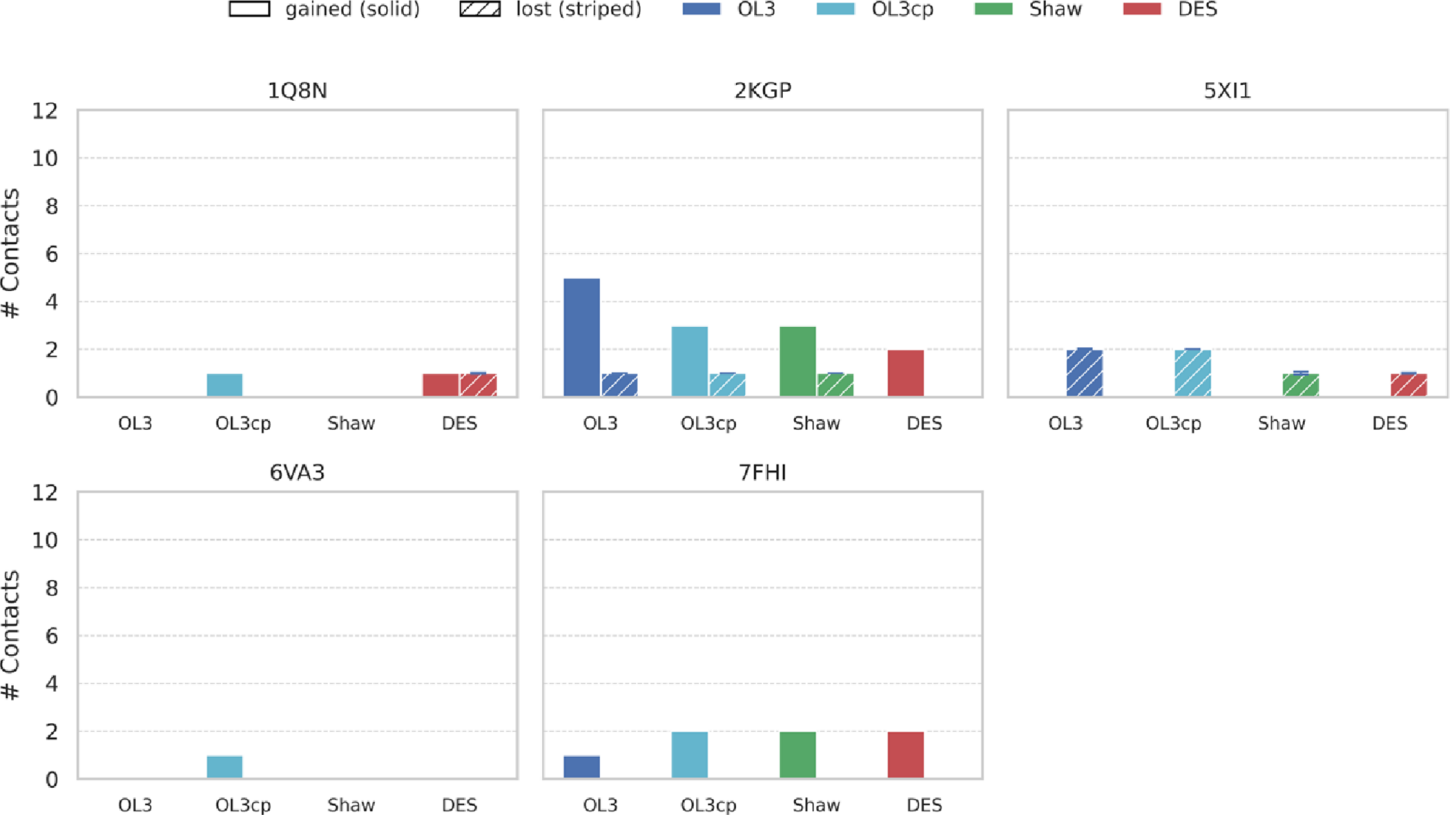
Changes in ligand–RNA contacts under different FFs for groove binders. Histograms showing the number of starting ligand contacts gained (full color) or lost (dashed color) (present in the experimental structure but broken in >10% of simulation frames). Bars: OL3 (dark blue), OL3cp (light blue), Shaw (green), DES-AMBER (red); PDB IDs above panels. Asterisks mark trajectories in which the ligand became unbound. For details on the contacts gained and lost, see Suppl. Figures S13–S14.

Thus, in the case of PDB ID 1Q8N, the RNA aptamer binds malachite green and the dye is intercalated via π–π stacking between a G·C base pair and a guanine-rich base quadruple/triple, enhancing complex stability. Electrostatic forces from the RNA’s phosphate backbone impose an asymmetric positive charge distribution on the ligand, prompting conformational adjustment in the dye. The ligand is stabilized not only by strong stacking with the bases but also by an increase in contacts with the RNA (see Figure 9A and Suppl. Figures S14–S16 and S17). Along the different simulations, the conformational changes look more like a rearrangement, without major loss of contacts/interactions, in particular using OL3 and OL3cp (see Suppl. Figure 14). DES does not lose intra-RNA interactions, but we could detect a strange deformation of the double-stranded RNA conformation (Figure 9A). The most important remark for the ligand site using Shaw FF is a temporary loss of the 8G-28C base pair at the intercalator site, where it interacts with the ligand, in contrast with the experimental observation. Unfortunately, we were unable to further assess the compatibility of this new arrangement with experimental data, as the NOE data were not available for comparison.

**Figure 9:**
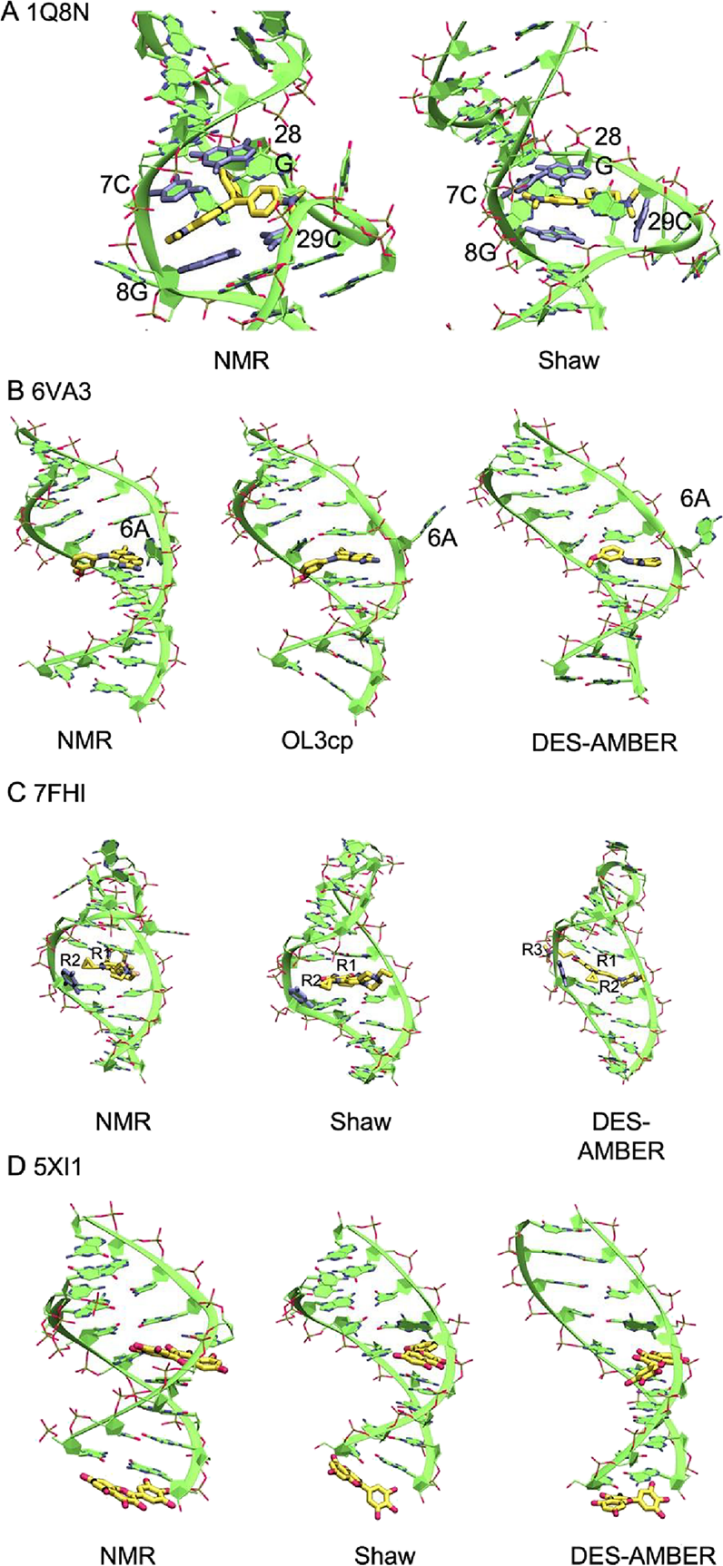
NMR structures and representative MD end-state snapshots for four RNA–ligand complexes. (A) 1Q8N: NMR structure was compared with Shaw. Residues 7C, 8G, 28G, and 29C are labeled to highlight local rearrangements in the ligand binding site. (B) 6VA3: NMR structure was compared with OL3cp and DES-AMBER. DES-AMBER shows a loss of helical shape, while OL3cp promotes deeper ligand intercalation near residue 6A. (C) 7FHI: NMR structure compared with Shaw and DES-AMBER. Both FFs enhance ligand intercalation and distort the local duplex groove. (D) 5XI1: NMR structure is compared with Shaw and DES-AMBER. The terminal intercalated ligand is not maintained, and helical geometry is disrupted.

In the case of PDB ID 6VA3 (see Figure 9B), a dibenzothiopene derivative targets a pre-mRNA by forming hydrogen bonds between its guanidinyl groups and the A-bulge’s closing nucleobases. Its aromatic portion also forms stacking interactions with adjacent base pairs. Using both OL3cp and DES-AMBER, the insertion of the ligand is enhanced, with the full opening of the A-bulge (see Figure 9B, Suppl. Figure S17). This behavior has been checked by comparing the result from this simulation and the restraints from the available NOEs data: the complete opening of the A-bulge is not compatible with experimental restraints, while the intercalation just involves the violation of two weak restraints not related to the adenine. However, as in the previous case, the main difference between these FFs is that DES-AMBER FF not only promotes the intercalation, but distorts the RNA helicity in disagreement with the experimental structure (Figure 9B). Using the other FFs, OL3 and Shaw, the unpaired A is more exposed to the solvent, but this does not involve major changes in the ligand binding, only local minimal variations in agreement with the experimental conformer.

The structural distortions associated with intercalation, at the expense of the groove and double-helical shape of the RNA, using the DES-AMBER FF are also evident in the PDB ID 7FHI and PDB ID 5XI1 structures (see Figure 9C,D and Suppl. Figure S17–S18).

In the PDB ID 7FHI complex, a fluoroquinolone derivative binds to an RNA hairpin into a bulged region, showing stacking, hydrogen bonding, and van der Waals contacts (see Figure 9C and Suppl. Figure S12). Using DES-AMBER, the interactions between the RNA and the ligand are consistent with the experimental NOE distance constraints. However, the *N*-methyl group (R3) of the ligand, which approaches C16 of the RNA, causes opening of the groove. This ligand–phosphate interaction is not observed in the NMR structure but is identified as a potential interaction based on the experimental data (Figure 9C). This movement results in a distortion of the helical shape, with the A-form being distorted compared to the experimental data. For this complex, Shaw FF also exhibits some anomalies with a mild deformation in the RNA structure. However, the ligand interactions are qualitatively consistent with the NMR data (Figure 9C). Only movement of the ligand is observed to maximize the stacking relative to the experimental structure. Using OL3 and OL3cp, the experimental key contacts and RNA are maintained along the simulations without major changes.

The PDB ID 5XI1 corresponds to the CAG-repeat duplex bound to two identical ligands, myricetin, which targets the 5′CAG/3′GAC RNA motif, whose major interaction involves intercalation and base stacking, including π–π stacking of its benzopyran ring and hydrogen bonding. One ligand is intercalated in the middle of the structure, and a ligand is bound at the terminal region. As before, using the DES-AMBER FF, the intercalation and two binding sites are maintained; however, the starting double-helical structure of the A-RNA is not preserved (see Figure 9D). Using Shaw FF, the A-RNA conformation is largely maintained, and the only significant discrepancy with the experimental complex is the partial loss and extreme movements of the terminal ligand along the simulation (see representative snapshots in Suppl. Figure S18), while the other is kept at the intercalation site. This second, terminal binding site has been experimentally detected only at higher myricetin concentrations and accordingly likely less stable and possibly a result of specific experimental conditions. OL3 and OL3cp are able to keep both ligands at their binding sites, and the complex structure is maintained along the simulations without major changes.

Finally, as previously observed for PDB ID 2KGP (Figure 2B), the main point to highlight is the systematic loss of hydrogen bonds within the RNA when using the OL3 FF, likely as a trade-off to maintain stacking interactions. In this complex, the mitoxantrone molecule, a highly cationic drug, primarily intercalates into the bulge region of tau RNA and forms hydrogen bonds with RNA bases and electrostatic interactions with the phosphate backbone via its side chains. This binding stabilizes the RNA structure, and no major variations in RNA–ligand contacts were detected across the different FFs. Furthermore, OL3cp both preserves the ligand binding and RNA structure but also improves the contacts with the phosphate groups and the ligand itself that have also been seen experimentally in some conformers (G5-ligand) (see Figure S2). No significant changes were observed with the other FFs, with only slight local variations that are consistent with those of the experimental conformer.

In general, intercalator complexes are well reproduced by different FFs, and systematic deviations are observed only for DES-AMBER, where the MD trajectories display a notable increase in major groove width compared with the experimental model (Supplementary Figure S6). This widening results in a more stretched duplex geometry and a less canonical helical conformation. However, due to the nature of the interactions of these ligands, longer simulation time could be needed to detect further distortions.

## Conclusions

In this study, we explored the use of molecular dynamics and various force fields, both widely used and recently refined, for studying RNA–ligand complexes. Regarding RNA, within the time scale of the simulations performed, we did not observe significant variations in intramolecular interactions or overall structural stability. The different force fields were generally able to preserve the experimental RNA conformation throughout the trajectories, with the newly refined force fields showing an improved overall performance. In particular, OL3cp demonstrated a better ability to maintain the RNA secondary structure, characterized by reduced fluctuations (RMSF), stable groove dimensions, canonical hydrogen bond patterns when not influenced by the ligand, and limiting contact loss. DES-AMBER showed strong consistency in preserving hydrogen-bond patterns across systems, though this often came at the expense of helical geometry, leading to groove widening. Across all simulations, twisting and puckering remained largely stable with only minor variations, and contact flickering did not differ substantially among the force fields. Notably, those FFs were able to relax some local distortions present in the refined experimental models, thereby improving the overall structural reliability of the simulated RNA ensembles. Focusing on ligand binding, our analysis shows that most force fields are prone to maintain and even increase stacking interactions, suggesting a generic problem in the way this interaction is introduced in FFs. The strength of the stacking interaction in current RNA force fields favors very stable trajectories for intercalators. Overall, OL3cp appears to be the most stable and balanced force field in our tests, offering improvements both in RNA structure maintenance and in ligand binding. Both Shaw and DES-AMBER can enhance the intercalation and binding but at the expense of strange distortions in the RNA conformation.

In cases where binding occurs through the grooves and electrostatics play a major role, the behavior is more variable and the interactions tend to be less stable. OL3cp force field shows some ability to improve electrostatic interactions compared to OL3 for groove-binding ligands. On the other hand, Shaw and especially DES-AMBER display weaker electrostatic contributions, which aligns with the observed loss of ligand binding or major structural rearrangements, incompatible with direct experimental observables.

Overall, despite recent improvements, such as those implemented in OL3cp, there is still room for further refinement, particularly in tuning the RNA–ligand interactions in addition to intra-RNA stability. In addition to improvements in RNA force fields, further standardization of ligand parametrization and QM protocols would be highly beneficial to refine RNA–ligand interaction studies and enhance the accuracy of binding predictions. Otherwise, rational design of RNA binders would be a very difficult task.

## Supplementary Material



## Notes

Details on selected RNA–ligand complexes and their respective simulations, including structures, sequences, and simulation analyses; RMSD, RMSF, and hydrogen-bond profiles, RNA helical parameters, contact maps (and corresponding contact-occupancy variation maps), and representative snapshots assessing RNA integrity and ligand interactions (PDF)
