# Supplementary material for: Success and Limitations of Current Force Fields for the Description of RNA–Ligand Complexes
Source: J Phys Chem B. 2025 Oct 31;129(45):11679–92. doi: 10.1021/acs.jpcb.5c05262 (PMC12621256; doi:10.1021/acs.jpcb.5c05262)
Supplement: Supplementary file 1 [file jp5c05262_si_001.pdf]

SUPPLEMENTARY INFORMATION

Success and limitations of current force fields for the description of RNA-ligand complexes

Paula Fernández Migens[1,2], Israel Serrano-Chacón[1], Modesto Orozco[1,2*], Federica Battistini[1,2*]

1.Institute for Research in Biomedicine (IRB Barcelona), Baldiri Reixac 10, Barcelona 08028, Spain
2.Departament de Bioquímica i Biomedicina, Facultat de Biologia, Universitat de Barcelona, Avgda Diagonal 647, Barcelona 08028, Spain

PDB ID	3D and 2D images with sequence	Technique employed	# of structures deposited	RMSD (Å)	Resolution (Å)
1EI2	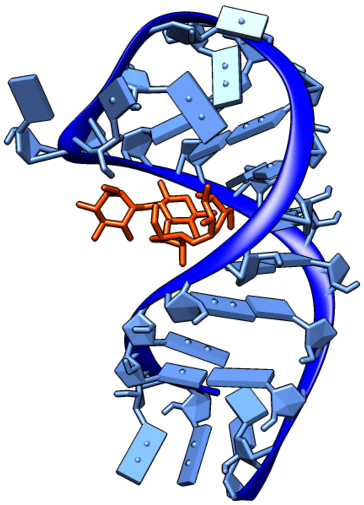<br>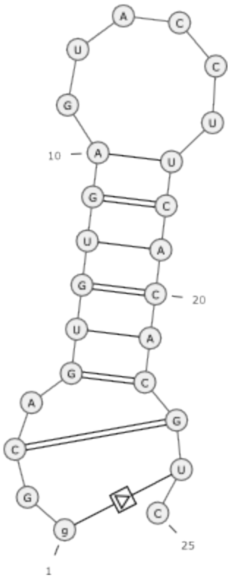<br><br>5’gGCAGUGUGAGUACCUUCACACGUC 3’	NMR	17	5.43 ± 1.2	-

1NTA	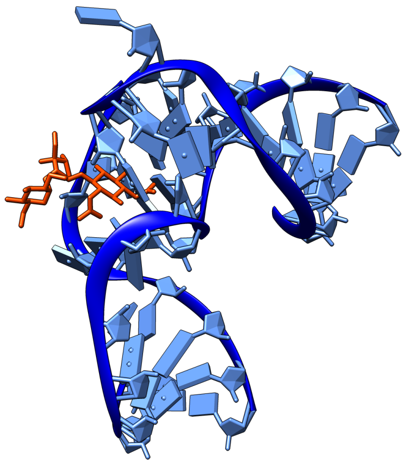<br>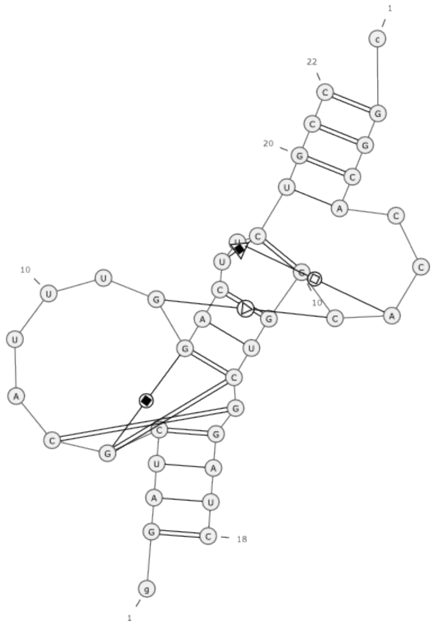<br>5’gGAUCGCAUUUGGACUUCUGCC 3’<br>5’cGGCACCACGGUCGGAUC 3’	X-ray	1	-	2.9

1Q8N	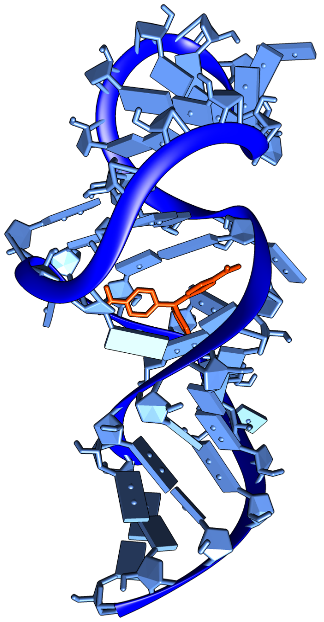<br>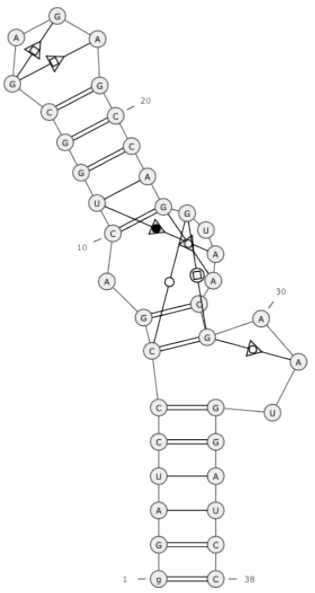<br><br>5’gGAUCCCGACUGGCGAGAGCCAGGUAAC-GAAUGGAUCC 3’	NMR	25	0.73 ± 0.12	-

1UUI	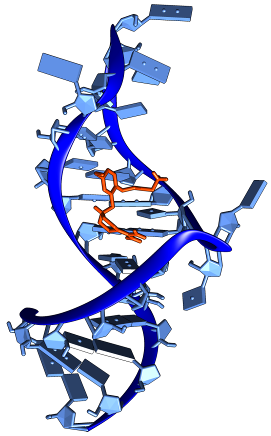<br>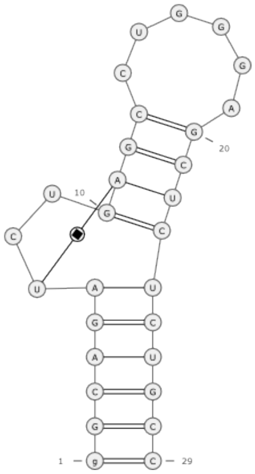<br>5’gGCAGACUGAGCCUGGGAGCCU-CUGCC 3’	NMR	1	-	-
2KGP	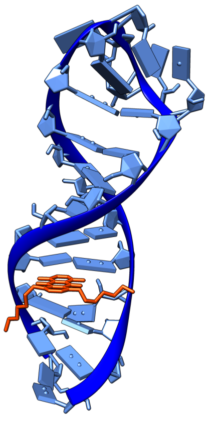	NMR	10	1.9 ± 0.70	-

	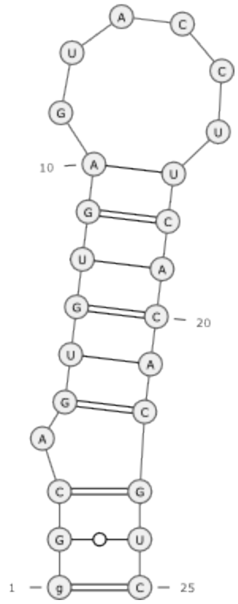 5’ gGCAGUGAGUACCUUCACACGUC 3’				
2L94	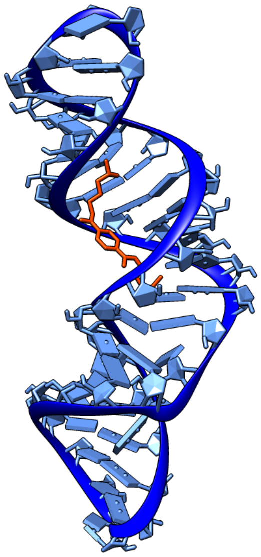	NMR	10	1.1 ± 0.48	-

	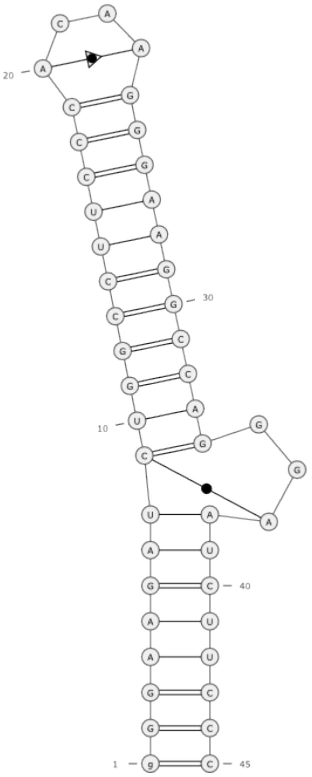 5’gGGAAGAU-CUGGCCUUCCCACAAGGGAAGGCCAGGGAAUCUUCCC 3’				
5XI1	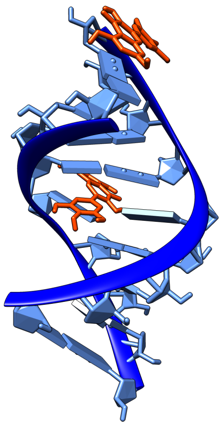	NMR	10	0.043 ± 0.026	-

	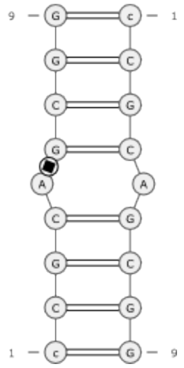 5’cCGCAGCGG 3’<br>5’cCGCAGCGG 3’				
6HMO	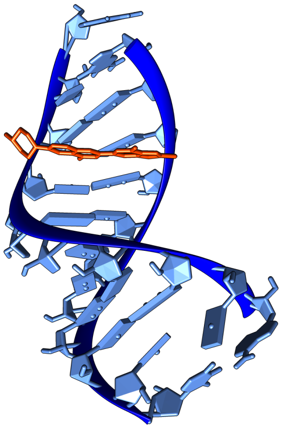	NMR	20	2.11 ± 0.99	-

	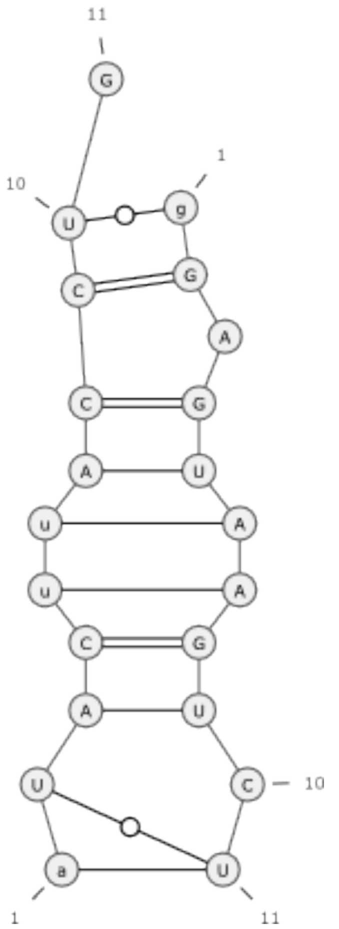<br>5’ aUACuuACCUG 3’<br>5’ gGAGUAAGUCU 3’				
6VA3	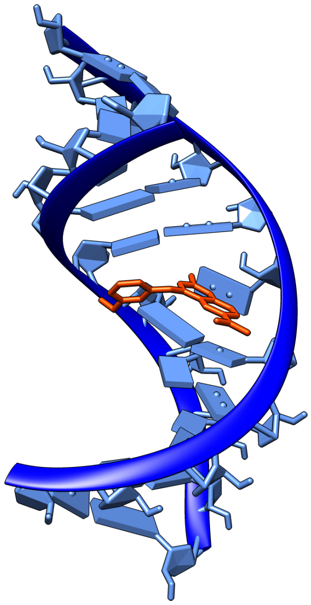	NMR	20	1.3 ± 0.37	-

	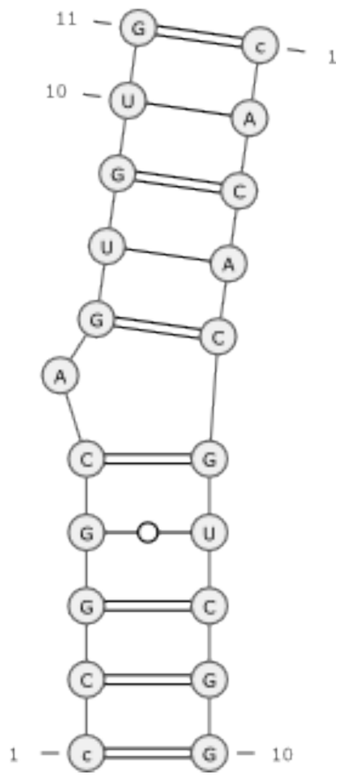<br>5'cCGGCAGUGUG 3'<br>5' cACACGUCGG 3'				
7FHI	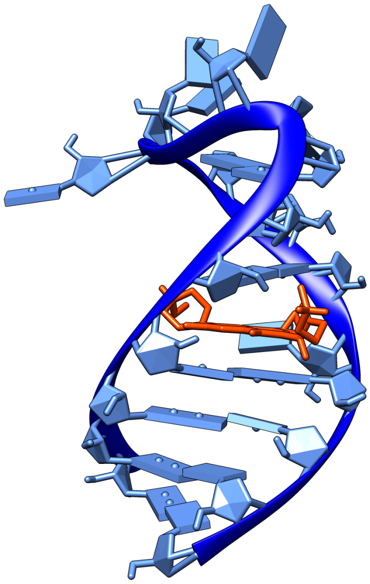	NMR	11	1.27 ± 0.99	-

	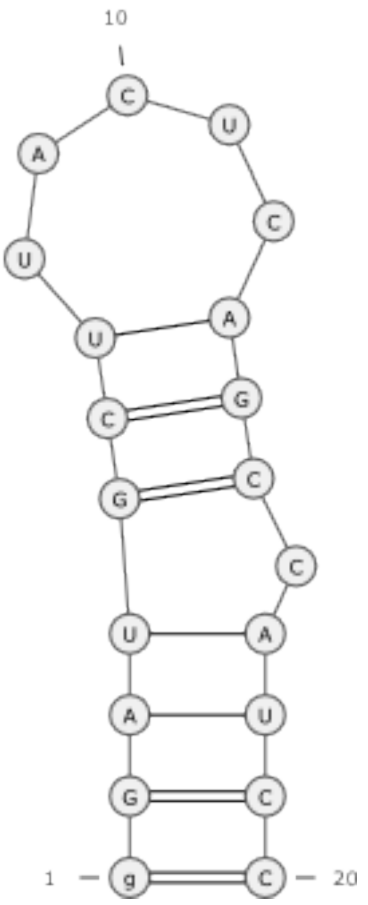<br>5’gGAUGCUUACUCAGCCAUCC 3’				

Supplementary Table S1. Representation of the selected structures (3D and 2D with sequence), complex and ligand and experimental details. Note that 2D representation was created using http://rnapdbee.cs.put.poznan.pl/ and base-pairs identified by 3DNA-DSSR. Non-canonical bps annotated in graphical representation. Structural elements identified treating pseudoknots as paired residues. Symbols following VARNA-based procedure.

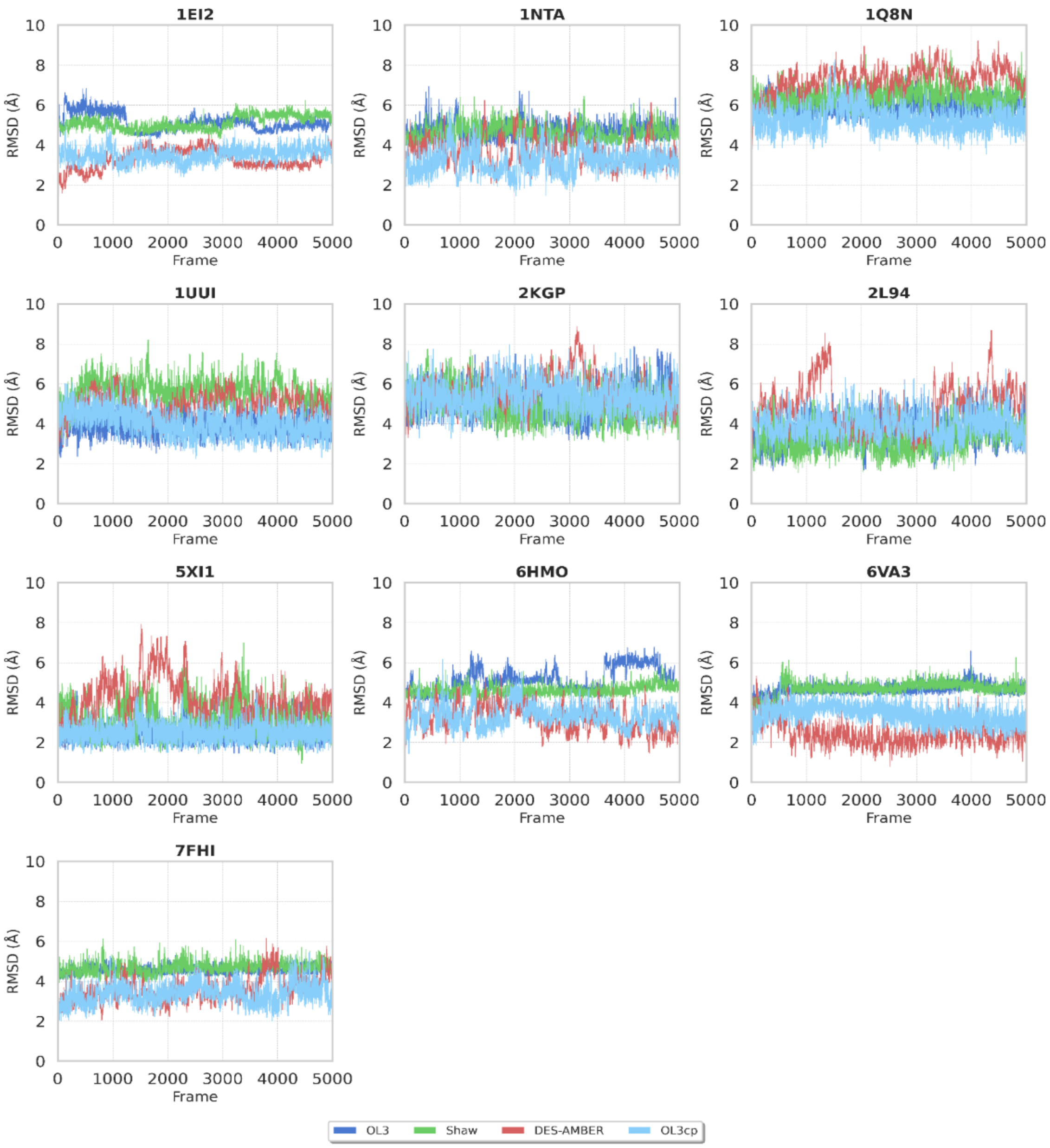

Supplementary Figure S1. RMSD relative to the initial experimental structure of RNA structures over the trajectory using four force fields: OL3 (dark blue), Shaw (green), OL3cp (sky blue), and DES-AMBER (red).

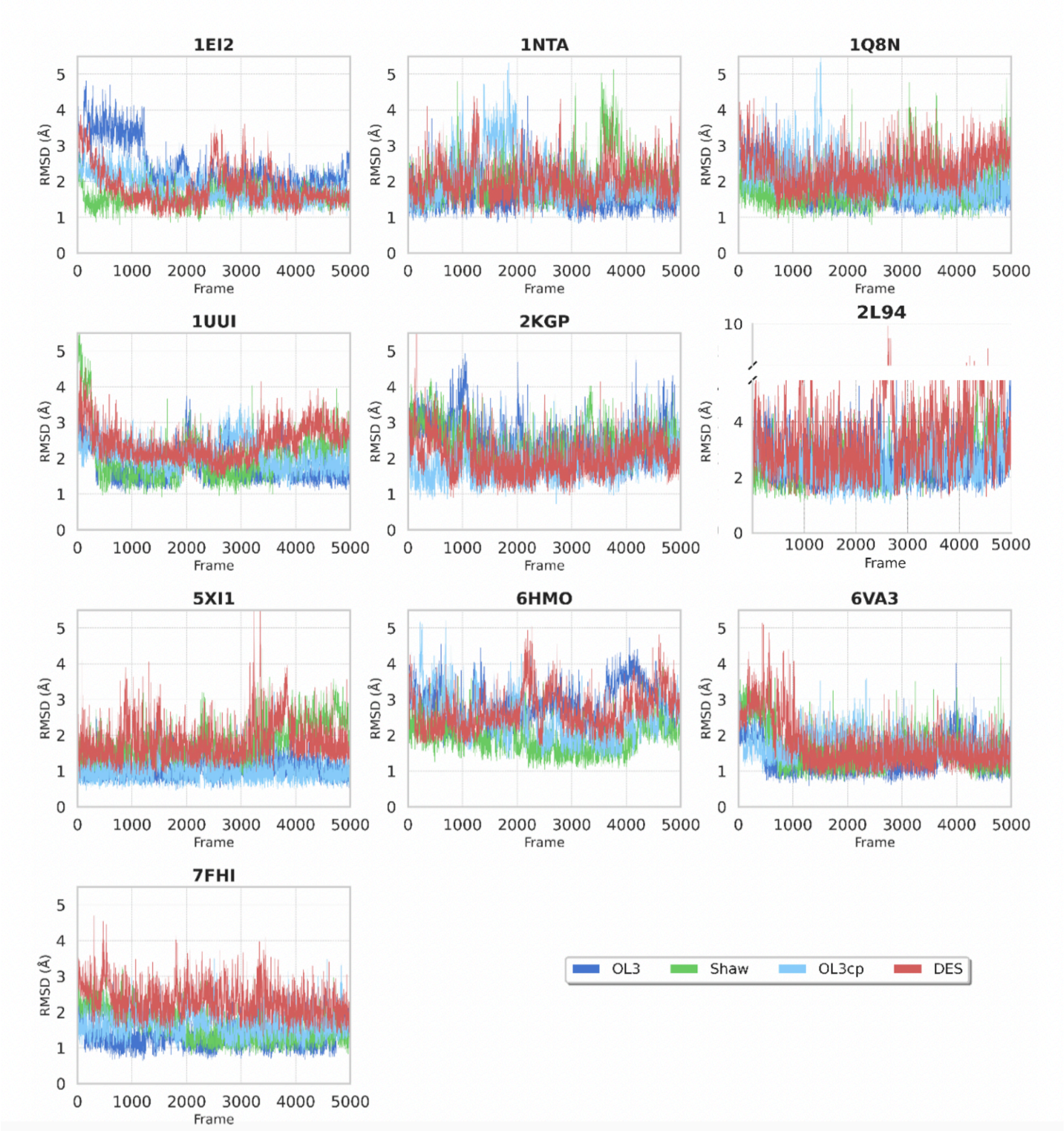

Supplementary Figure S2. RMSD (Å) of each RNA in the complex relative to the average structure for each trajectory. Simulations were performed with OL3 (dark blue), Shaw (green), OL3cp (light blue), and DES-AMBER (red).

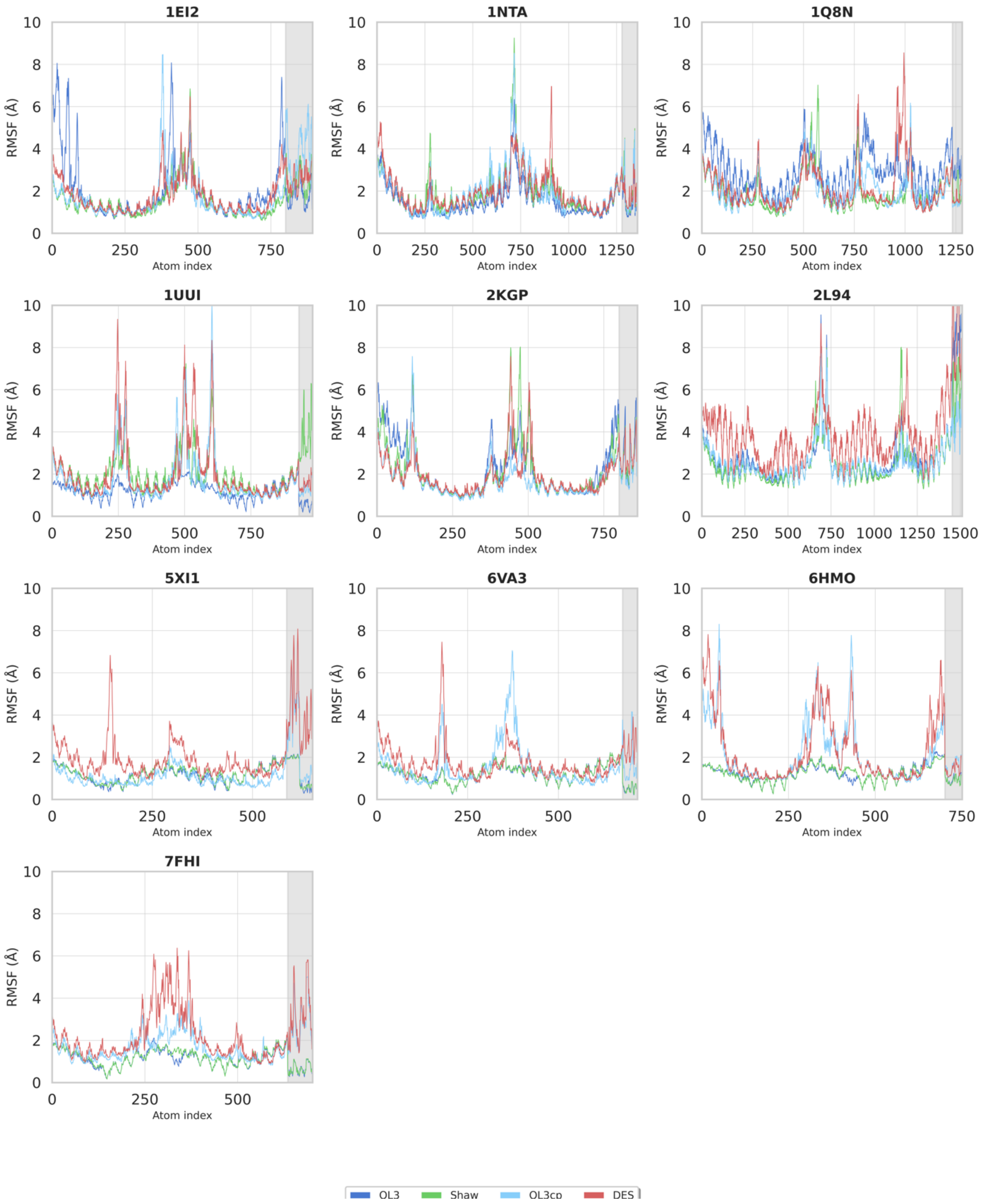

Supplementary Figure S3. RMSF (Å) for RNA and ligand (grey shadow) for each simulation. Simulations were performed with OL3 (dark blue), Shaw (green), OL3cp (light blue), and DES-AMBER (red).

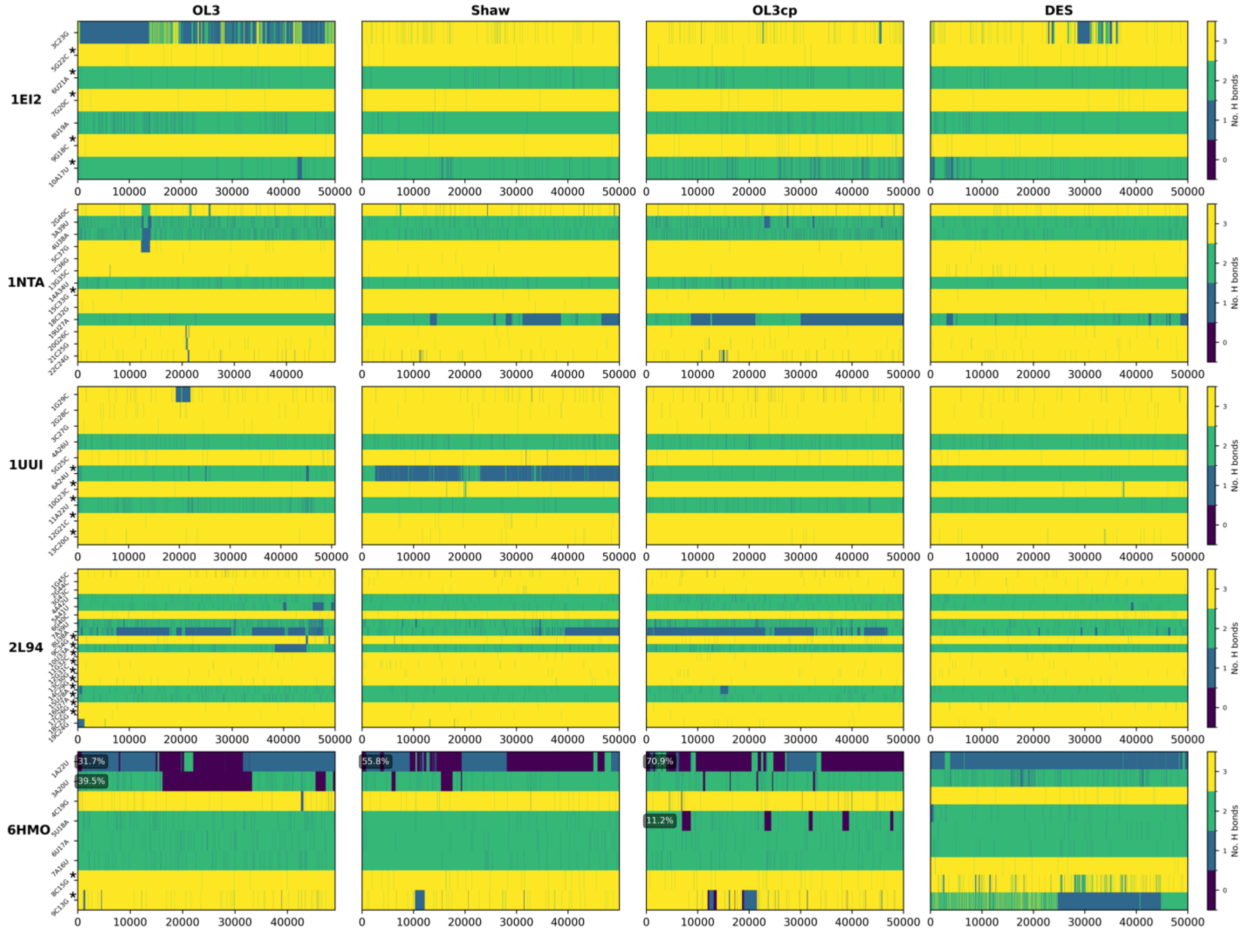
OL3
Shaw
OL3cp
DES
1EI2
1NTA
1UUI
2L94
6HMO
No. H bonds
31.7%
39.5%
55.8%
70.9%
11.2%

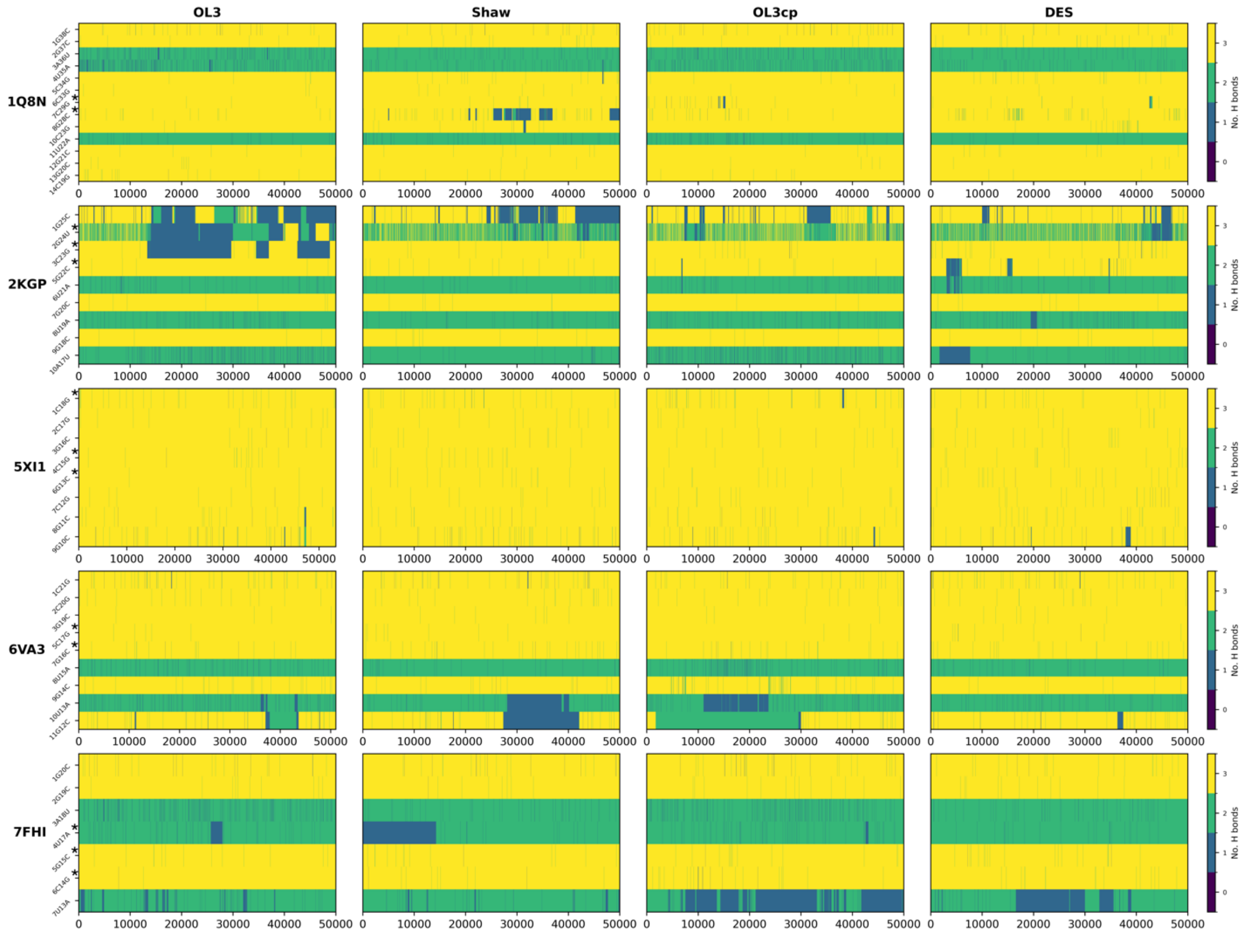

Supplementary Figure S4. Time evolution of hydrogen bonds present between each base pair (canonical and non) in each respective simulation respect to the initial experimental structure. Top panels groove binders, bottom panels intercalator cases. The star next to the base pair step indicates that the base or bases are interacting with the ligand.

Loss (%)		OL3			Shaw			OL3cp			DES		
Structure	Base pairing	1H	2H	3H	1H	2H	3H	1H	2H	3H	1H	2H	3H
**1EI2**	All	2.8 ± 3.6	2.9 ± 6.7	10 ± 26	2.2 ± 3.0	0.2 ± 0.3	-	3.7 ± 3.8	0.4 ± 0.7	0.2 ± 0.4	2.4 ± 2.3	1.2 ± 2.0	3.2 ± 8.5
	AU	2.9 ± 3.5	0.6 ± 1.0	-	1.6 ± 1.0	0.0 ± 0.1	-	5.0 ± 4.2	0.3 ± 0.4	-	2.6 ± 2.4	1.3 ± 2.2	-
	GC	2.7 ± 4.1	4.5 ± 9.0	17 ± 35	2.6 ± 4.1	0.2 ± 0.4	0.0 ± 0.1	2.6 ± 3.8	0.5 ± 0.8	0.3 ± 0.6	2.2 ± 2.7	1.1 ± 2.1	5.7 ± 11
**1NTA**	All	2.4 ± 4.1	6.6 ± 18.2	0.1 ± 0.3	1.3 ± 1.3	5.1 ± 16	-	2.4 ± 4.0	6.9 ± 21	-	1.3 ± 1.3	2.3 ± 7.5	-
	AU	6.3 ± 5.0	17 ± 29	-	2.5 ± 1.3	13 ± 27	-	6.2 ± 4.7	18 ± 34	-	2.1 ± 1.1	6.3 ± 12	-
	GC	0.2 ± 0.2	0.6 ± 1.3	0.2 ± 0.4	0.6 ± 0.7	0.0	-	0.2 ± 0.2	0.0 ± 0.1	0.0	0.8 ± 1.3	0.1 ± 0.2	0.0
**1Q8N**	All	2.1 ± 3.8	0.3 ± 0.5	-	1.5 ± 1.2	0.3 ± 0.5	2.1 ± 6.8	2.1 ± 2.9	0.1 ± 0.2	0.3 ± 0.6	1.4 ± 1.2	0.2 ± 0.3	0.0 ± 0.1
	AU	7.0 ± 5.5	0.9 ± 0.8	-	2.6 ± 0.6	0.2 ± 0.2	-	5.9 ± 3.4	0.2 ± 0.1	-	1.9 ± 1.2	-	-
	GC	0.5 ± 0.4	0.1 ± 0.2	0.0	1.1 ± 1.1	0.3 ± 0.6	2.7 ± 7.9	0.8 ± 1.0	0.1 ± 0.2	0.3 ± 0.7	1.3 ± 1.2	0.3 ± 0.3	0.0
**1UUI**	All	1.5 ± 1.5	0.3 ± 0.6	-	10 ± 25	1.7 ± 4.3	-	1.0 ± 1.0	0.1 ± 0.1	-	0.9 ± 0.7	0.1 ± 0.1	-
	AU	2.7 ± 1.6	0.6 ± 1.0	-	25 ± 40	4.1 ± 7.1	-	2.0 ± 1.1	0.1 ± 0.1	-	1.2 ± 1.1	0.1 ± 0.1	-
	GC	0.8 ± 1.0	0.1 ± 0.1	0.0	0.8 ± 0.2	0.2 ± 0.3	0.0	0.4 ± 0.3	0.1 ± 0.1	-	0.8 ± 0.5	0.1 ± 0.2	-
**2KGP**	All	2.1 ± 3.0	0.2 ± 0.4	7.5 ± 21	1.1 ± 0.7	0.1 ± 0.1	-	2.6 ± 3.7	0.2 ± 0.2	-	1.5 ± 1.2	3.1 ± 5.2	0.9 ± 2.6
	AU	4.9 ± 3.7	0.2 ± 0.2	-	1.8 ± 0.3	0.1 ± 0.0	-	5.7 ± 5.1	0.2 ± 0.3	-	2.1 ± 0.9	7.4 ± 6.9	-
	GC	0.6 ± 0.2	0.3 ± 0.6	15 ± 30	0.9 ± 0.3	0.1 ± 0.0	-	1.0 ± 0.5	0.2 ± 0.2	-	1.5 ± 1.2	0.6 ± 0.6	1.9 ± 3.7
**2L94**	All	2.4 ± 3.6	5.1 ± 15.3	0.0	1.5 ± 1.8	1.4 ± 5.3	-	2.1 ± 2.6	4.6 ± 17	-	1.2 ± 0.8	0.3 ± 0.9	-
	AU	5.2 ± 4.4	12.1 ± 22.9	-	2.6 ± 2.4	3.4 ± 8.2	-	4.3 ± 2.9	11 ± 27	-	1.7 ± 1.0	0.7 ± 1.3	-
	GC	0.5 ± 0.2	0.1 ± 0.3	0.0	0.8 ± 0.4	0.1 ± 0.0	0.0	0.5 ± 0.3	0.1 ± 0.1	-	0.9 ± 0.3	0.1 ± 0.0	-
**5XI1**	All	0.7 ± 0.3	0.2 ± 0.2	0.1 ± 0.3	1.0 ± 0.6	0.3 ± 0.2	-	0.6 ± 0.1	0.2 ± 0.3	-	1.3 ± 0.6	0.2 ± 0.1	-
	AU	-	-	-	-	-	-	-	-	-	-	-	-
	GC	0.7 ± 0.3	0.2 ± 0.2	0.1 ± 0.3	1.0 ± 0.6	0.3 ± 0.2	0.0	0.6 ± 0.1	0.2 ± 0.3	0.0	1.3 ± 0.6	0.2 ± 0.1	0.0
**6HMO**	All	1.3 ± 1.4	6.0 ± 14.9	0.1 ± 0.1	1.0 ± 0.8	1.4 ± 2.7	0.3 ± 0.8	1.1 ± 1.0	1.3 ± 1.9	1.0 ± 2.6	1.9 ± 2.1	2.1 ± 4.3	0.2 ± 0.4
	AU	3.2 ± 0.4	19.8 ± 28.0	-	1.4 ± 0.6	3.6 ± 5.1	-	2.2 ± 0.8	1.5 ± 2.1	-	3.5 ± 3.4	1.4 ± 1.9	-
	GC	0.9 ± 0.7	0.7 ± 0.6	0.1 ± 0.1	1.4 ± 0.6	0.8 ± 1.2	0.7 ± 1.3	1.1 ± 0.3	2.0 ± 2.6	2.4 ± 3.9	2.1 ± 1.4	4.0 ± 6.6	0.4 ± 0.7
**6VA3**	All	2.2 ± 2.5	0.6 ± 1.2	-	1.3 ± 0.8	3.6 ± 9.1	-	5.1 ± 8.8	1.0 ± 1.7	-	1.6 ± 0.9	0.1 ± 0.1	-
	AU	5.6 ± 2.2	1.7 ± 2.3	-	1.8 ± 0.3	12 ± 17	-	15 ± 12	2.2 ± 3.0	-	2.6 ± 1.0	0.1 ± 0.0	-
	GC	0.8 ± 0.6	0.2 ± 0.2	0.0	1.1 ± 0.8	0.2 ± 0.3	0.0	0.9 ± 0.7	0.6 ± 1.0	0.0	1.3 ± 0.7	0.1 ± 0.1	0.0
**7FHI**	All	4.0 ± 4.3	1.3 ± 1.8	-	1.9 ± 1.5	5.0 ± 11	-	4.4 ± 4.9	9.9 ± 22	-	1.5 ± 0.5	5.8 ± 13	-

	AU	7.4 ± 3.5	2.4 ± 2.0	-	2.7 ± 1.9	9.8 ± 16	-	7.9 ± 4.8	19 ± 32	-	1.8 ± 0.6	11 ± 19	-
	GC	0.7 ± 0.2	0.1 ± 0.1	0.0	1.1 ± 0.3	0.2 ± 0.2	0.0	0.8 ± 0.1	0.3 ± 0.1	0.0	1.3 ± 0.1	0.2 ± 0.2	0.0

Loss (%)	OL3			Shaw			OL3cp			DES		
Base pairing	1H	2H	3H	1H	2H	3H	1H	2H	3H	1H	2H	3H
All	2.2 ± 0.7	3.2 ± 4.3	2.7 ± 5.0	2.1 ± 2.2	2.9 ± 3.5	0.9 ± 1.4	2.3 ± 1.3	3.4 ± 4.1	1.0 ± 2.0	1.5 ± 0.4	2.5 ± 3.9	0.6 ± 1.1
AU	5.4 ± 1.7	8.6 ± 13	-	4.7 ± 7.8	8.7 ± 11	-	6.1 ± 4.0	9.5 ± 11	-	2.1 ± 0.6	6.7 ± 11	-
GC	0.9 ± 0.7	0.9 ± 1.4	4.7 ± 9.0	1.2 ± 0.5	0.7 ± 0.9	1.4 ± 2.1	1.0 ± 0.6	0.6 ± 0.6	1.5 ± 2.6	1.4 ± 0.5	0.8 ± 1.2	1.1 ± 1.9

Supplementary Table S2. Hydrogen bond loss (% in time) compared to the starting experimental structure. Top table data considering the base pairs (all) and divided by CG and AT pairing for each PDB and FFs considered. Bottom table average over each PDB for each FFs.

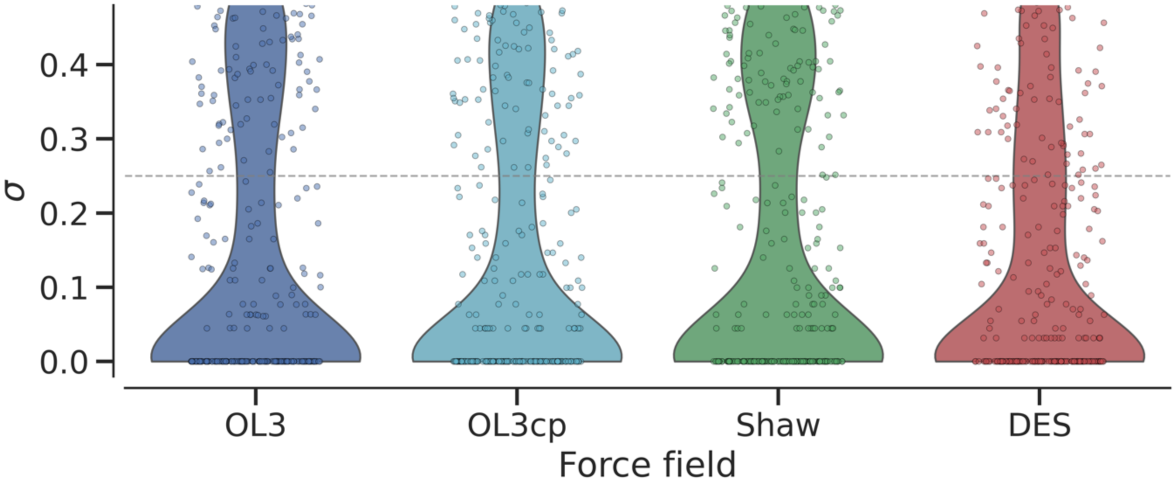

Supplementary Figure S5. RNA-RNA contact stability across the trajectory: σ is the standard deviation of the frame-by-frame occupancy of RNA–RNA heavy-atom contact: a value of 0 means the contact is maintained throughout the trajectory, whereas values that rise toward 0.5 reflect increasingly “flickery” contacts that break and re-form repeatedly. Colours correspond to the four FFs: OL3 (dark blue), OL3cp (light blue), Shaw (green), and DES-AMBER (red).

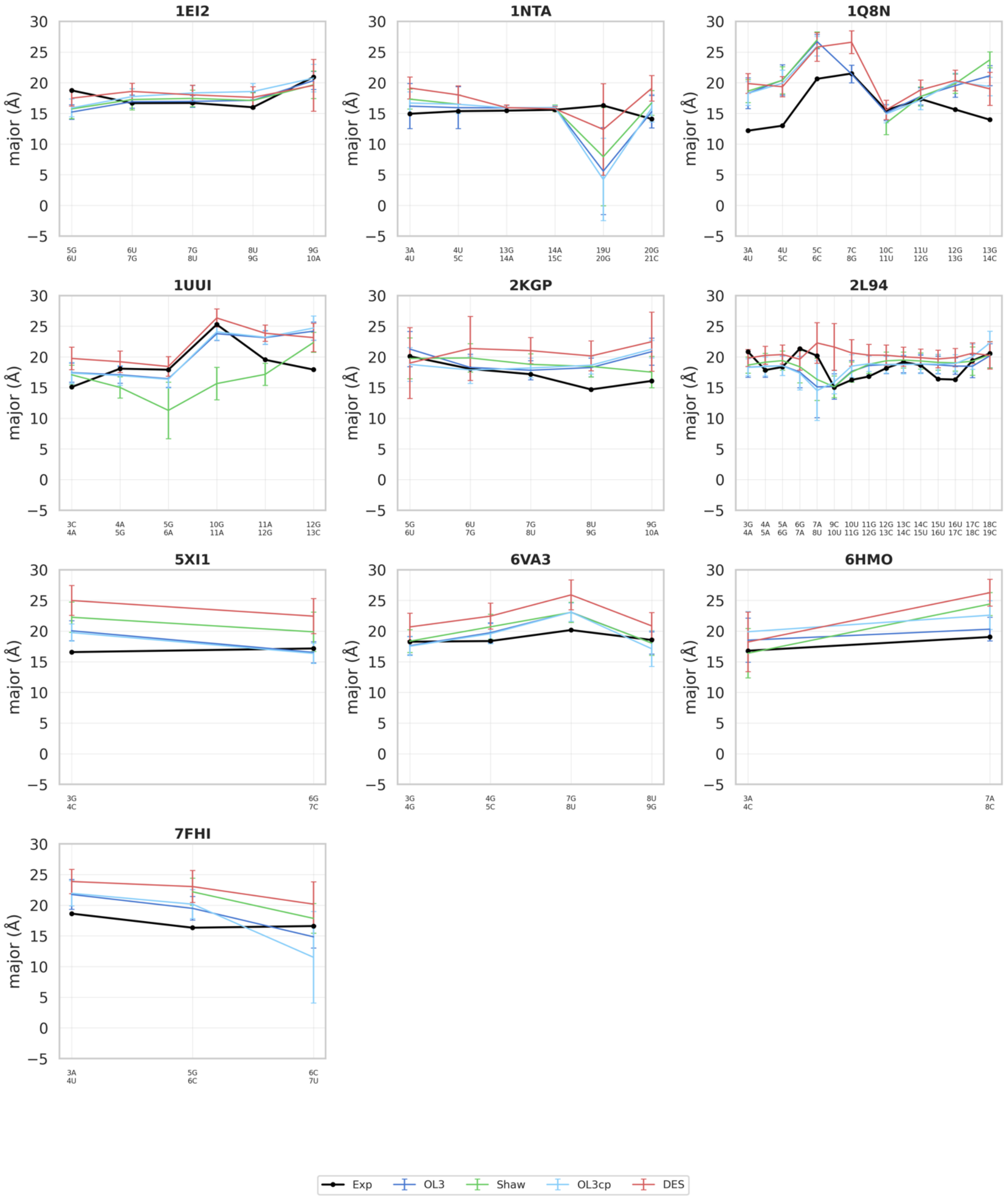

Supplementary Figure S6. Major Groove width (Å) along the RNA for each simulation. Average values with standard deviations correspond to simulations performed with OL3 (dark blue), Shaw (green), OL3cp (light blue), and DES-AMBER (red) and the reference starting structure (in black).

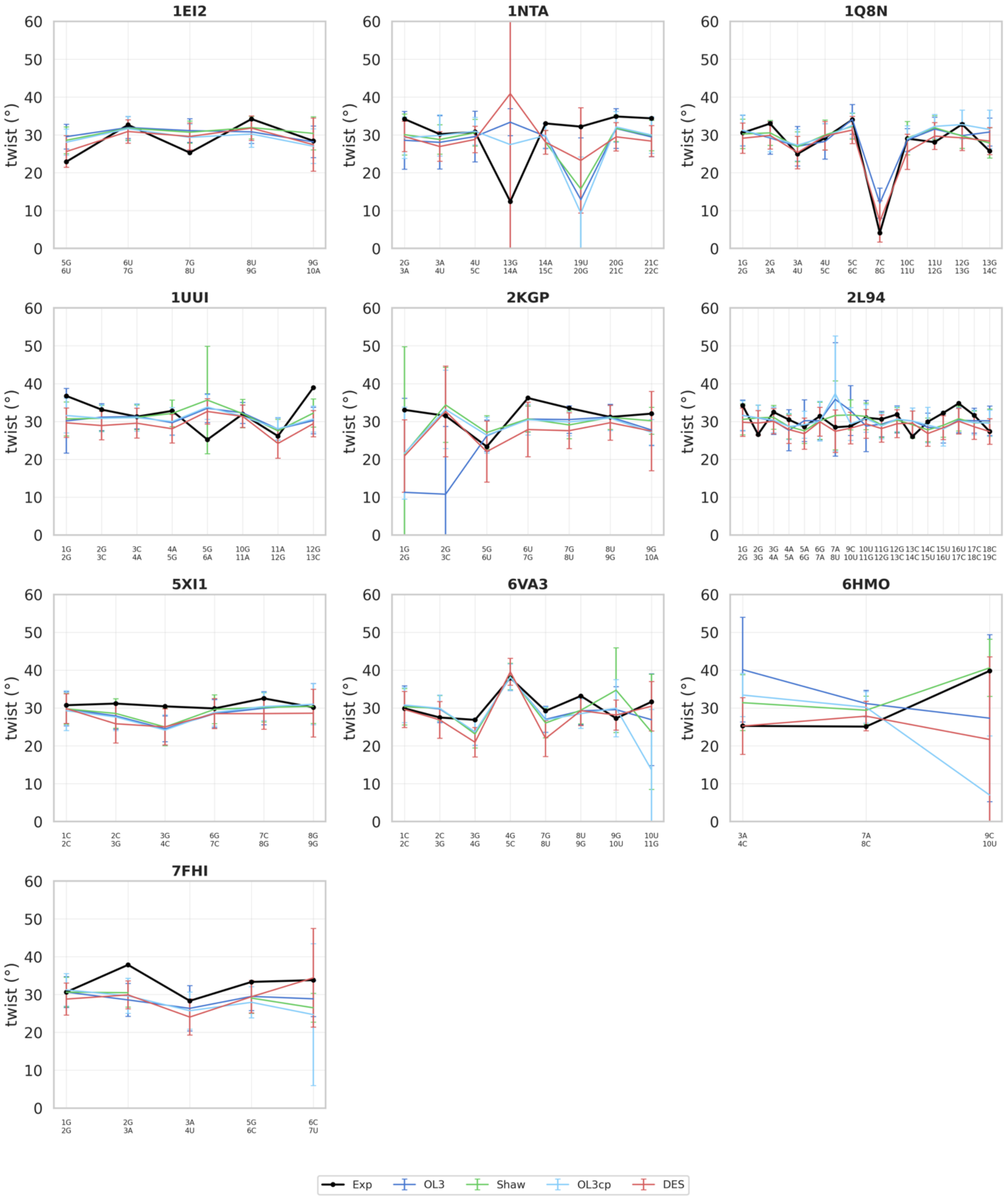

Supplementary Figure S7. Twist (degrees) along the RNA for each simulation. Average values with standard deviations correspond to simulations performed with OL3 (dark blue), Shaw (green), OL3cp (light blue), and DES-AMBER (red) and the reference starting structure (in black).

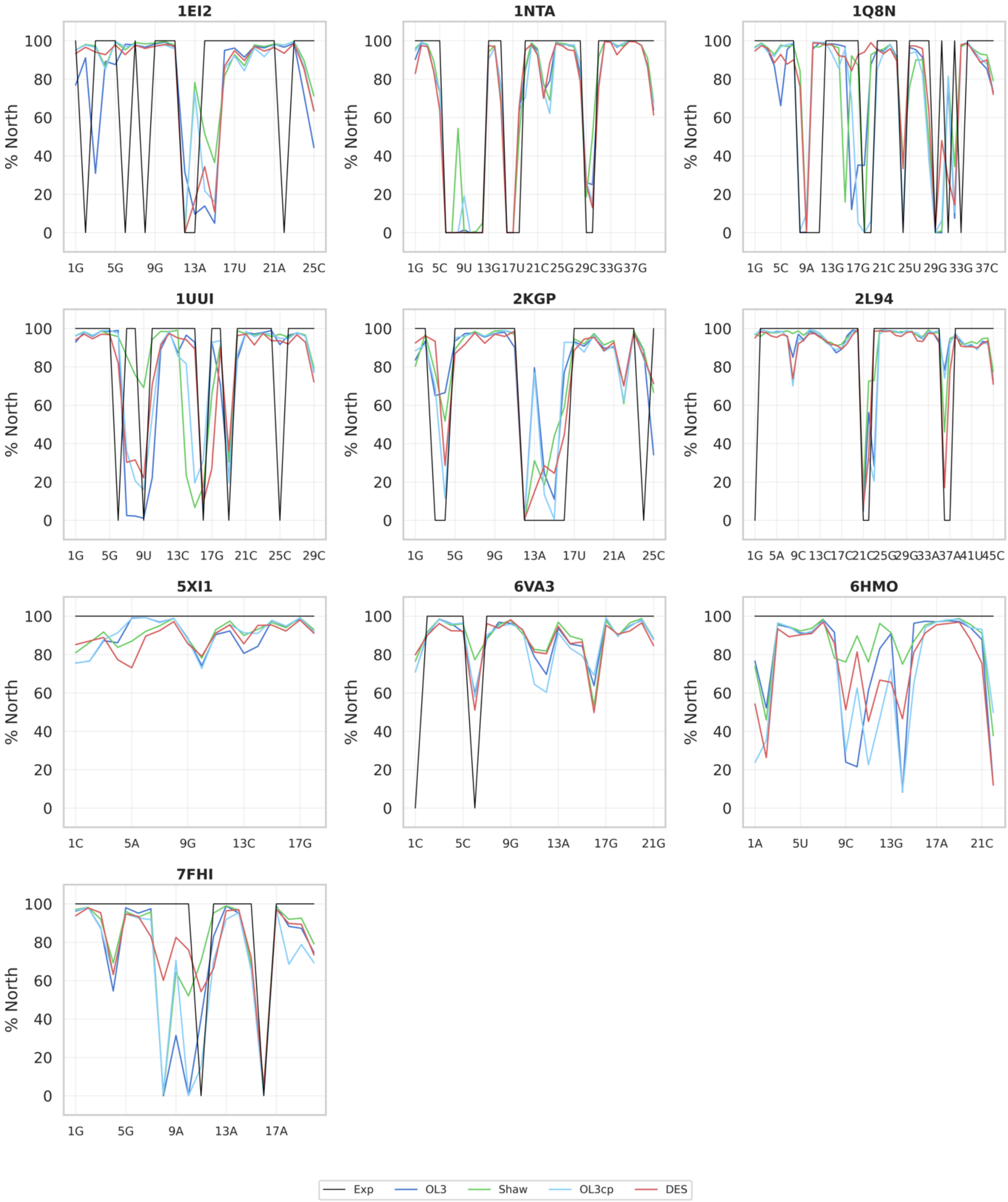

Supplementary Figure S8. Percentage of north puckering along the RNA for each simulation, performed with OL3 (dark blue), Shaw (green), OL3cp (light blue), and DES-AMBER (red) respectively and the reference starting structure (in black).

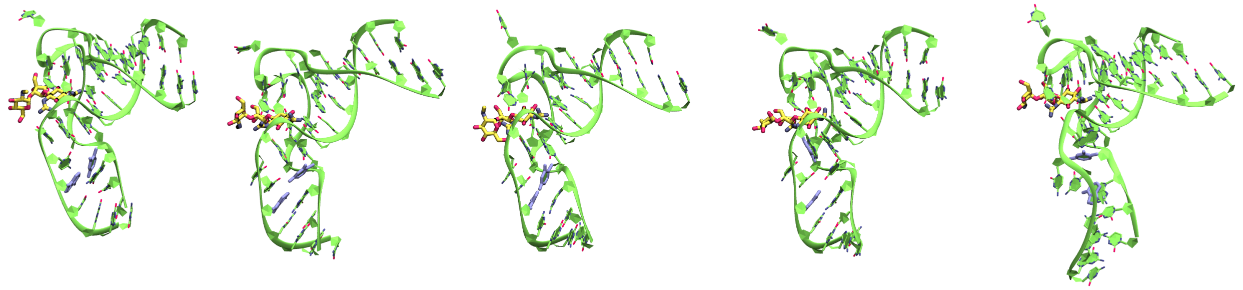

X-Ray OL3 Shaw OL3cp DES

Supplementary Figure S9 1NTA: Evolution of the U19-A27 base pair across force fields. Representative conformations of the RNA-ligand complex from MD simulations using four RNA force fields (OL3, Shaw, OL3cp, and DES) compared to the experimental structure (X-Ray). The U19-A27 base pair is highlighted in purple.

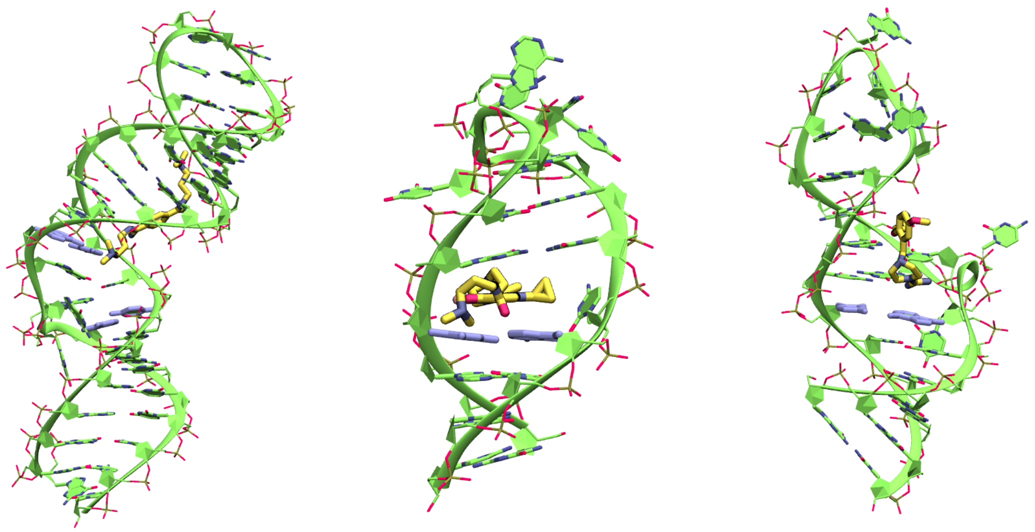

2L94 7FHI 1UUI

Supplementary Figure S10. Base-pairing interactions at the ligand-binding interface in experimental RNA-ligand complexes. Structures of three RNA-ligand complexes from the PDB: 2L94 (left), 7FHI (center), and 1UUI (right). Key base pairs at the binding interface are shown in purple: U8-A39 and U10-A33 in 2L94, U4-A17 in 7FHI, and A6-U24 in 1UUI (renumbered from A6–U23 in the original structure).

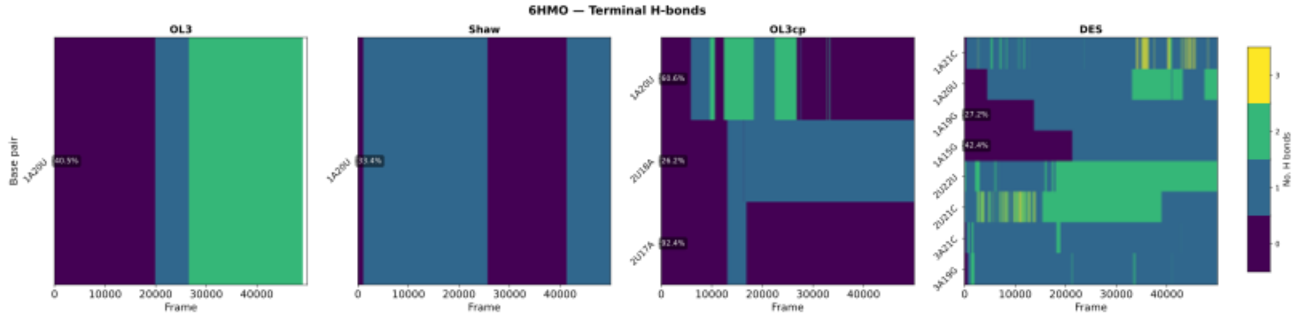

Supplementary Figure S11. Hydrogen bonds formed along the simulations for the terminal part of 6HMO structure.

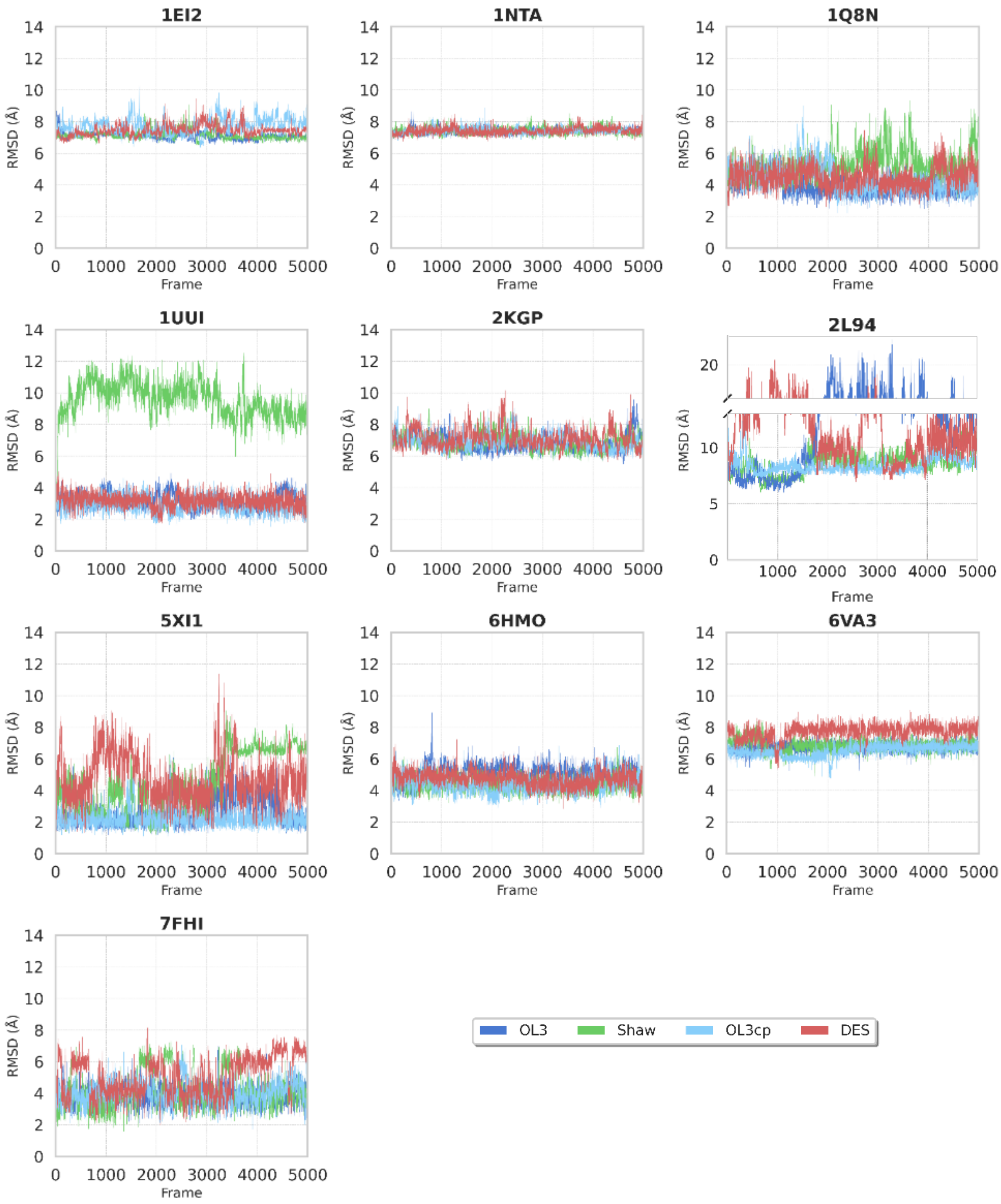

Supplementary Figure S12. LoRMSD for each RNA–ligand system over the trajectory. Trajectories were aligned to the experimental nucleic structure before computing the ligand RMSD. Colors: OL3 (dark blue), Shaw (green), OL3cp (sky blue), DES-AMBER (red).

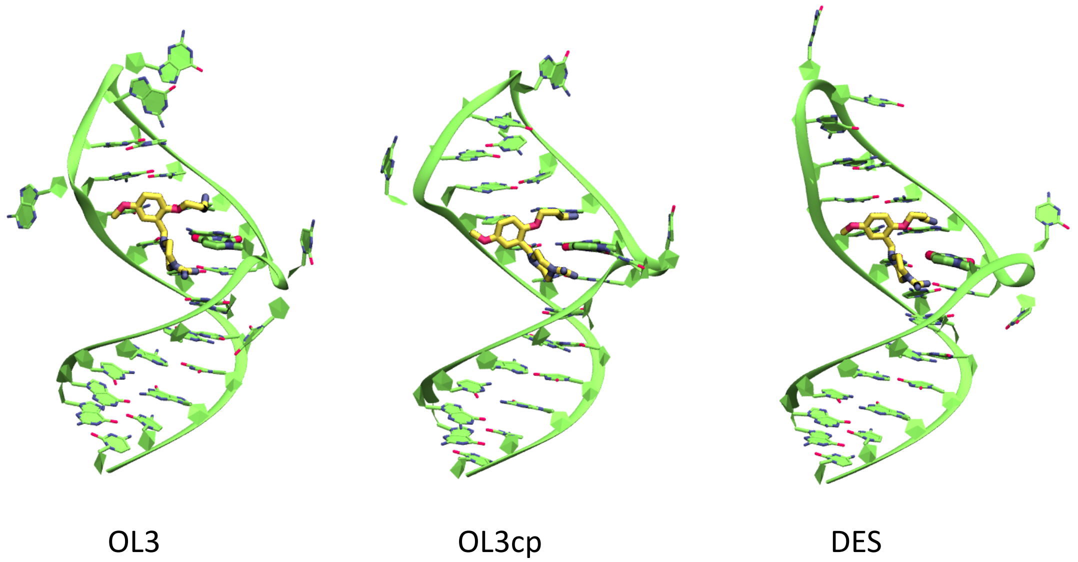

Supplementary Figure S13. Representative conformations from MD simulations of the 1UUI ligand complex using OL3, OL3cp, and DES force fields. RNA–ligand binding preserved across these three force fields, engaging key electrostatic "hot spot" interactions around U7.

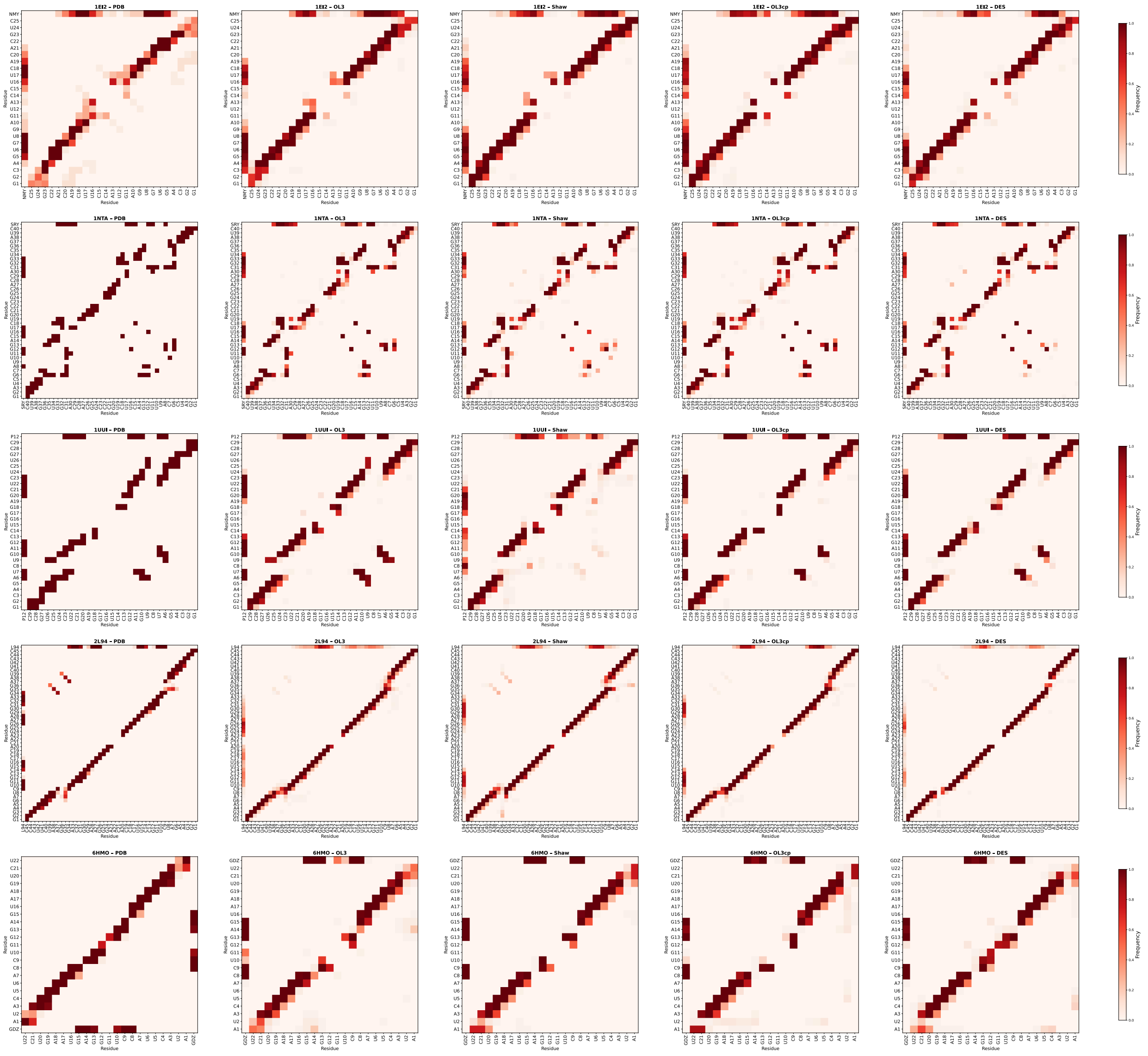

1EI2 – PDB
1EI2 – OL3
1EI2 – Shaw
1EI2 – OL3cp
1EI2 – DES
1NTA – PDB
1NTA – OL3
1NTA – Shaw
1NTA – OL3cp
1NTA – DES
1UUI – PDB
1UUI – OL3
1UUI – Shaw
1UUI – OL3cp
1UUI – DES
2L94 – PDB
2L94 – OL3
2L94 – Shaw
2L94 – OL3cp
2L94 – DES
6HMO – PDB
6HMO – OL3
6HMO – Shaw
6HMO – OL3cp
6HMO – DES
Residue
Frequency
0.0
0.2
0.4
0.6
0.8
1.0

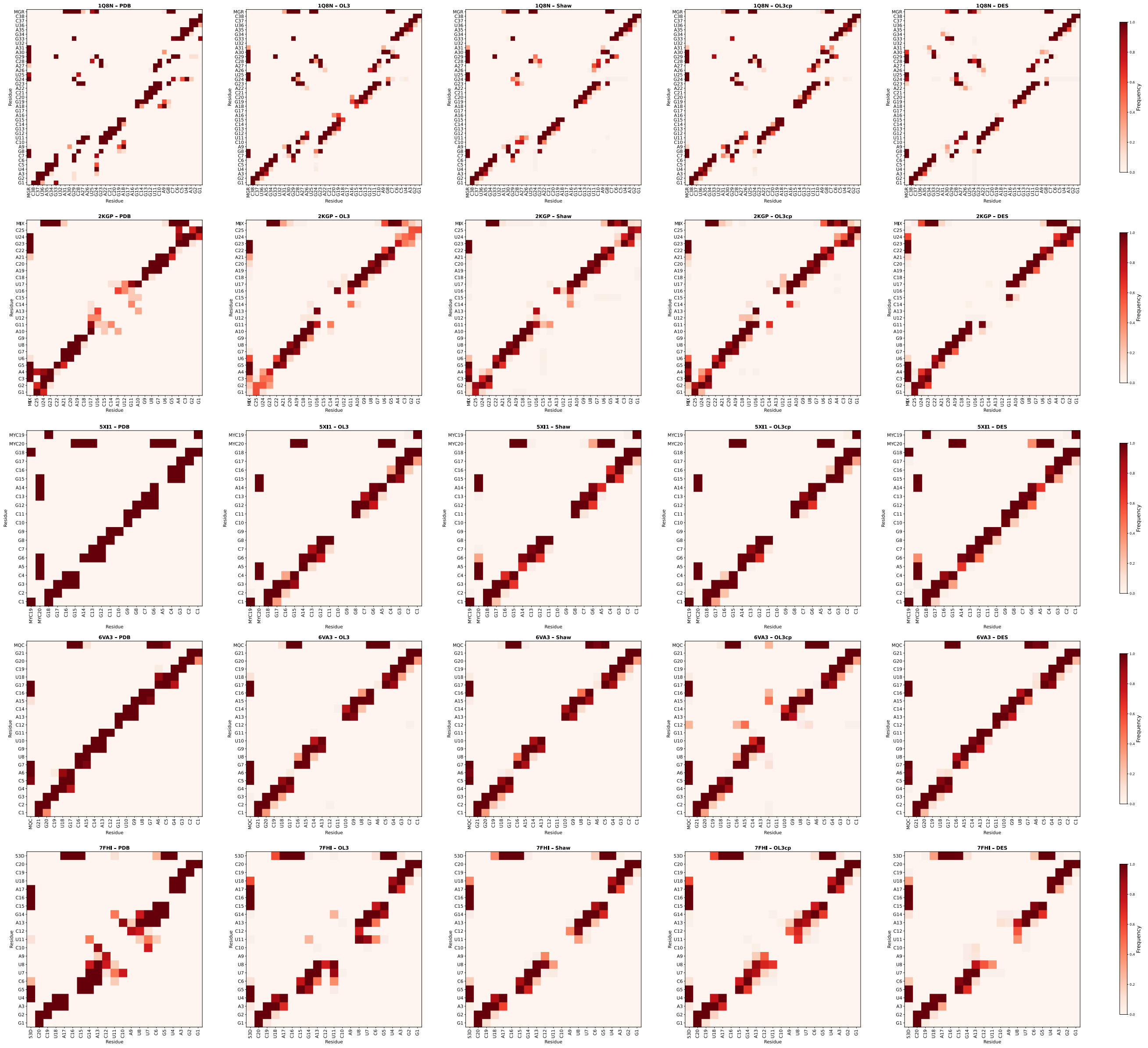

Supplementary Figure S14. Time-averaged contact maps for each RNA system. The PDB (ensemble) contact map is shown first (where multiple solution NMR models exist, values are averaged across models), followed by simulations with OL3, Shaw, OL3cp, and DES-AMBER. Matrix entries report the fraction of frames in which a residue pair is in contact (1: always; 0: never).

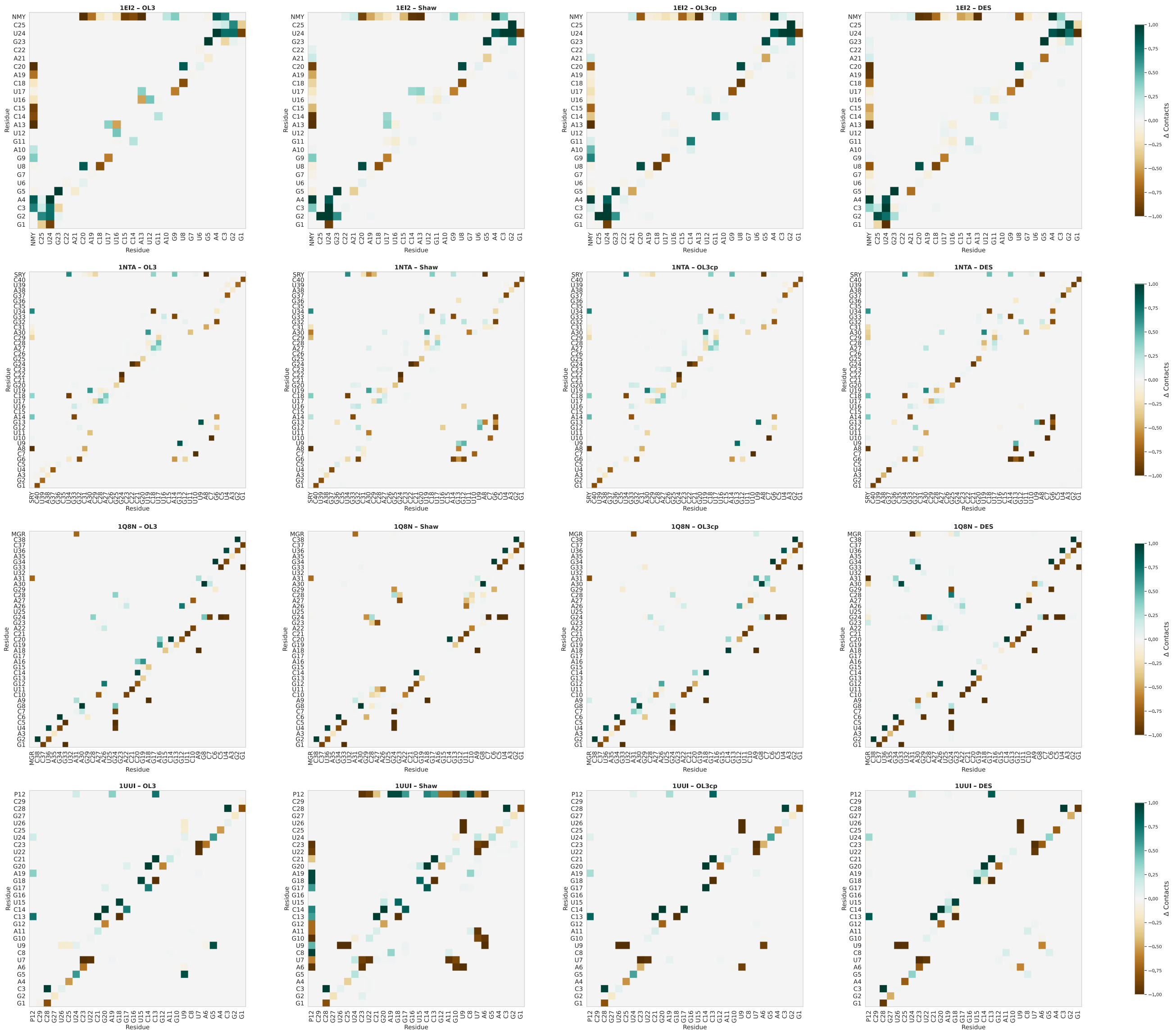
1EI2 – OL3
1EI2 – Shaw
1EI2 – OL3cp
1EI2 – DES
1NTA – OL3
1NTA – Shaw
1NTA – OL3cp
1NTA – DES
1Q8N – OL3
1Q8N – Shaw
1Q8N – OL3cp
1Q8N – DES
1UUI – OL3
1UUI – Shaw
1UUI – OL3cp
1UUI – DES
Residue
Δ Contacts
1,00
0,75
0,50
0,25
0,00
−0,25
−0,50
−0,75
−1,00

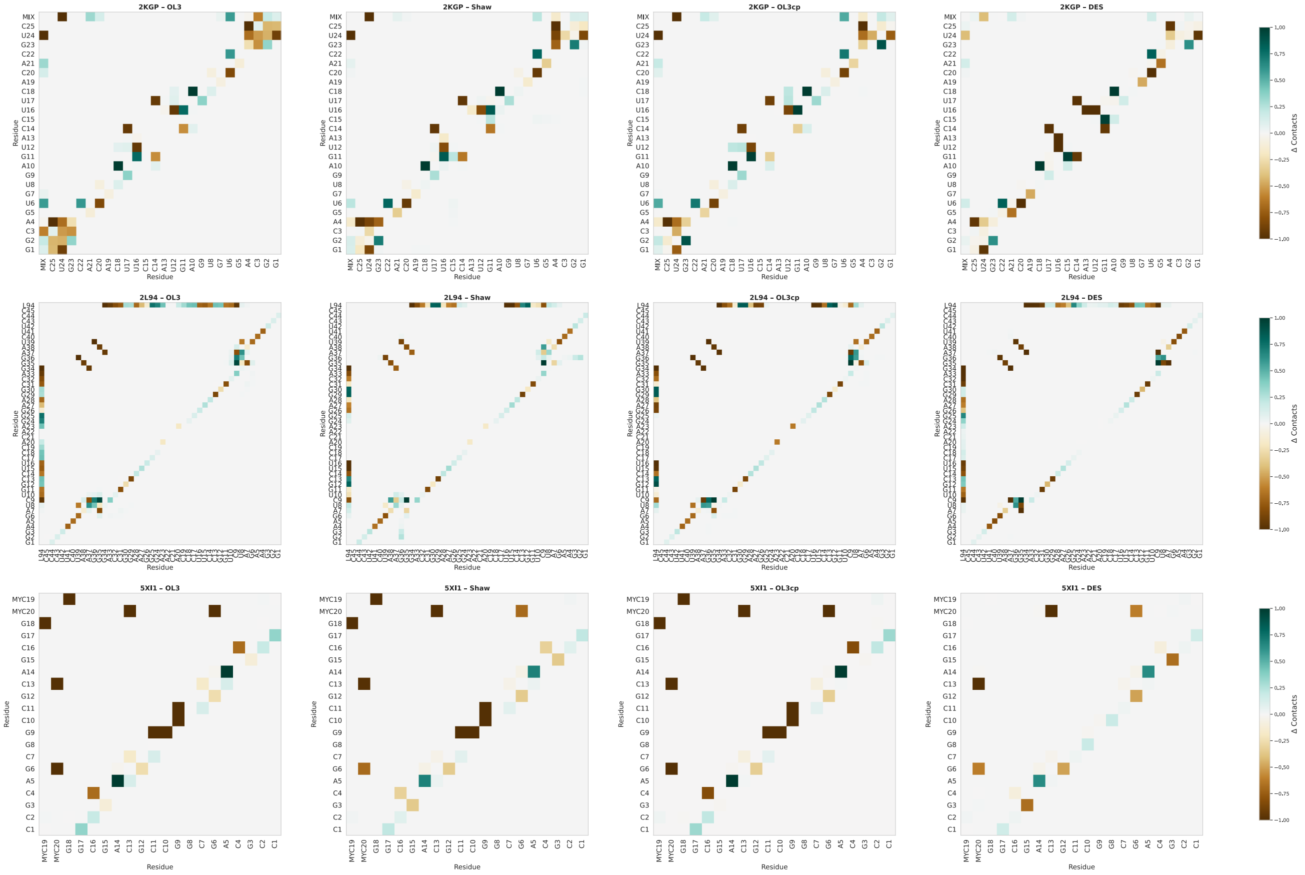
2KGP – OL3
2KGP – Shaw
2KGP – OL3cp
2KGP – DES
2L94 – OL3
2L94 – Shaw
2L94 – OL3cp
2L94 – DES
5XI1 – OL3
5XI1 – Shaw
5XI1 – OL3cp
5XI1 – DES
Residue
Δ Contacts

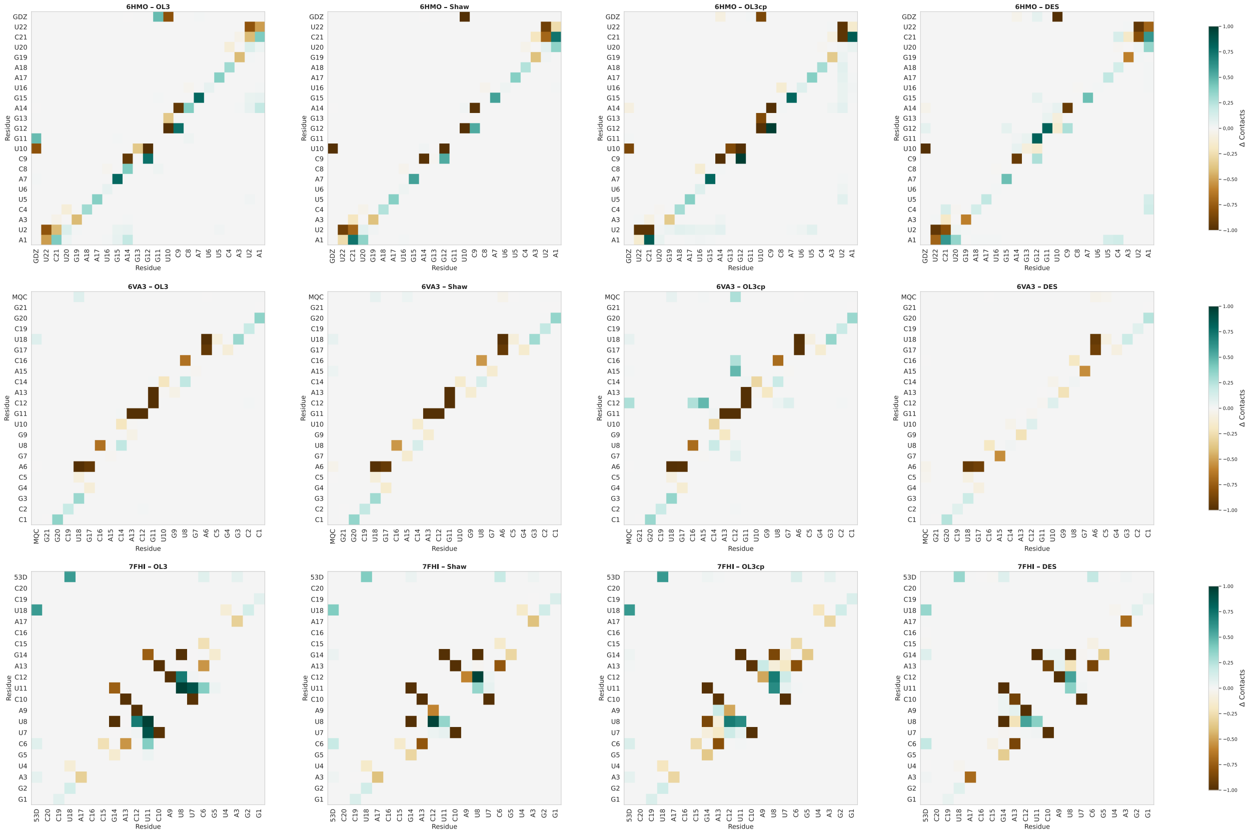

Supplementary Figure S15. Δ contact maps (simulation average contact map − starting experimental structure) for each RNA system and force field (OL3, Shaw, OL3cp, DES-AMBER). Matrix values report the change in time-averaged residue–residue contact probability relative to the initial structure. Positive Δ (green) denotes contacts gained; negative Δ (brown) denotes contacts lost.

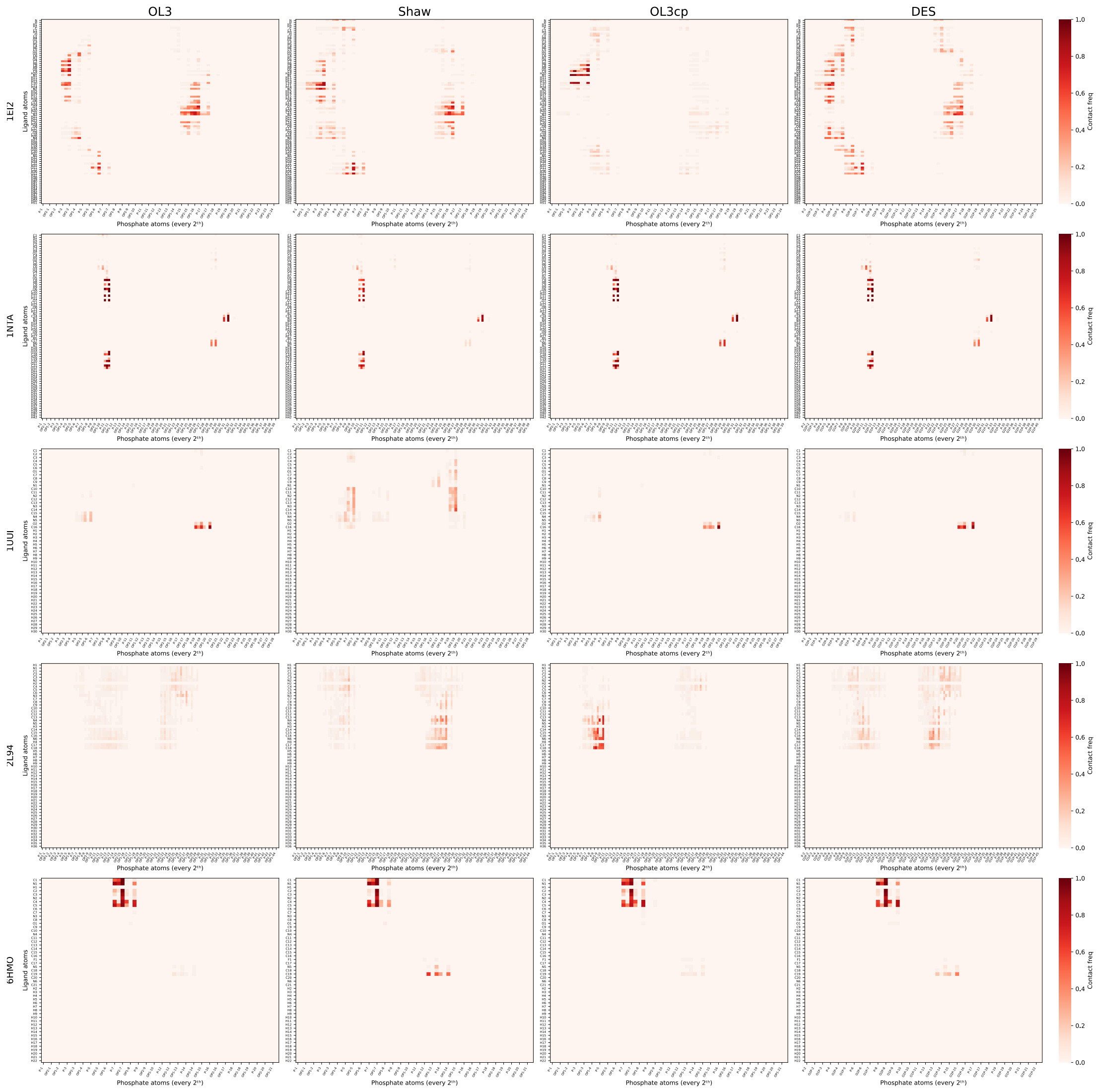

OL3
Shaw
OL3cp
DES
1EI2
1NTA
1UUI
2L94
6HMO
Ligand atoms
Phosphate atoms (every 2nd)
Contact freq
0.0
0.2
0.4
0.6
0.8
1.0

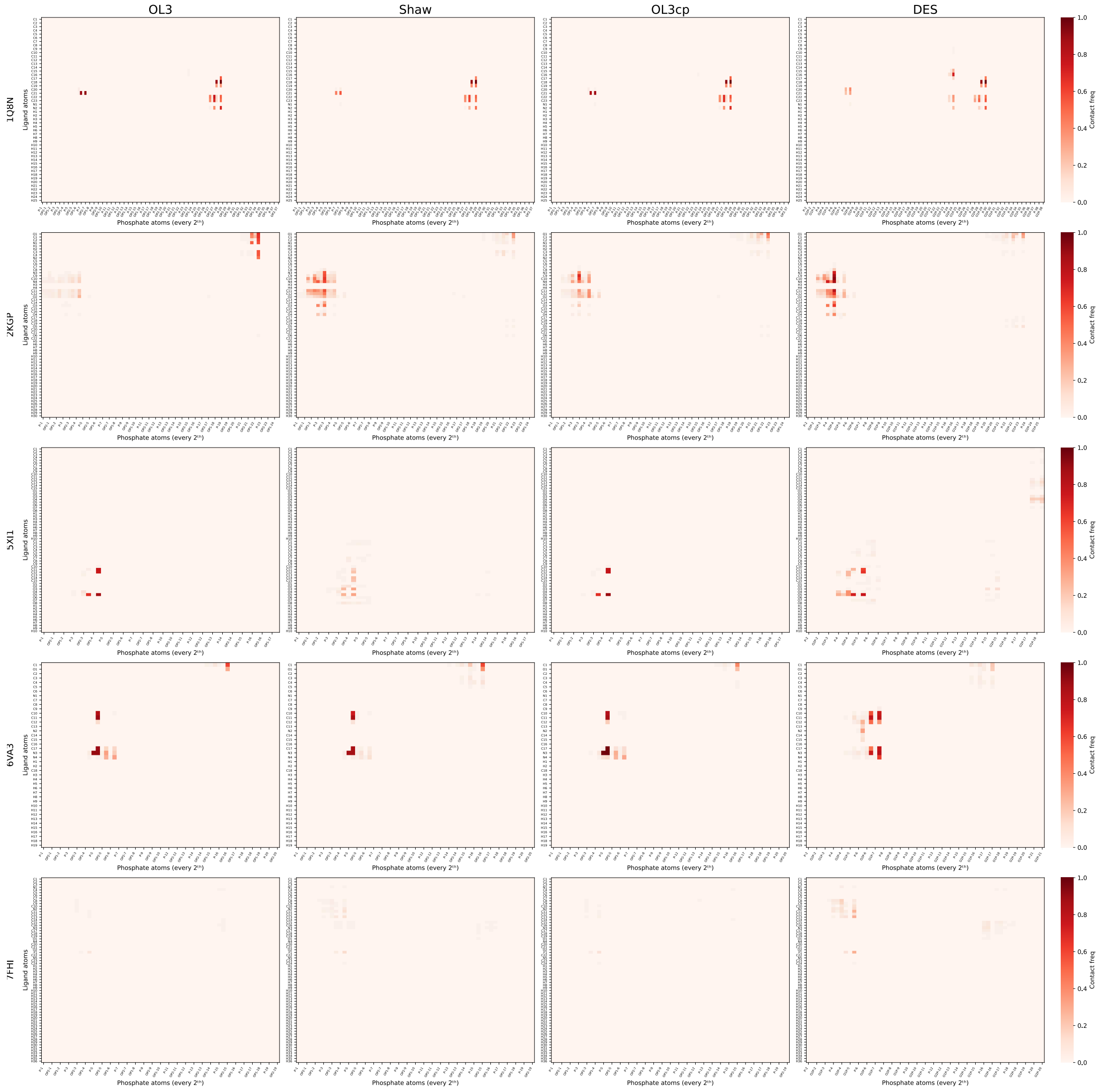

Supplementary Figure S16. Per-atom ligand–phosphate time-averaged contact maps for each RNA–ligand complex across force fields (OL3, Shaw, OL3cp, DES-AMBER). Within each panel, rows list ligand atoms; columns list RNA phosphate heavy atoms ordered along the sequence. Matrix values (0–1) give the fraction of simulation frames in which a ligand atom is within the contact cutoff of the corresponding phosphate atom (1 = always; 0 = never).

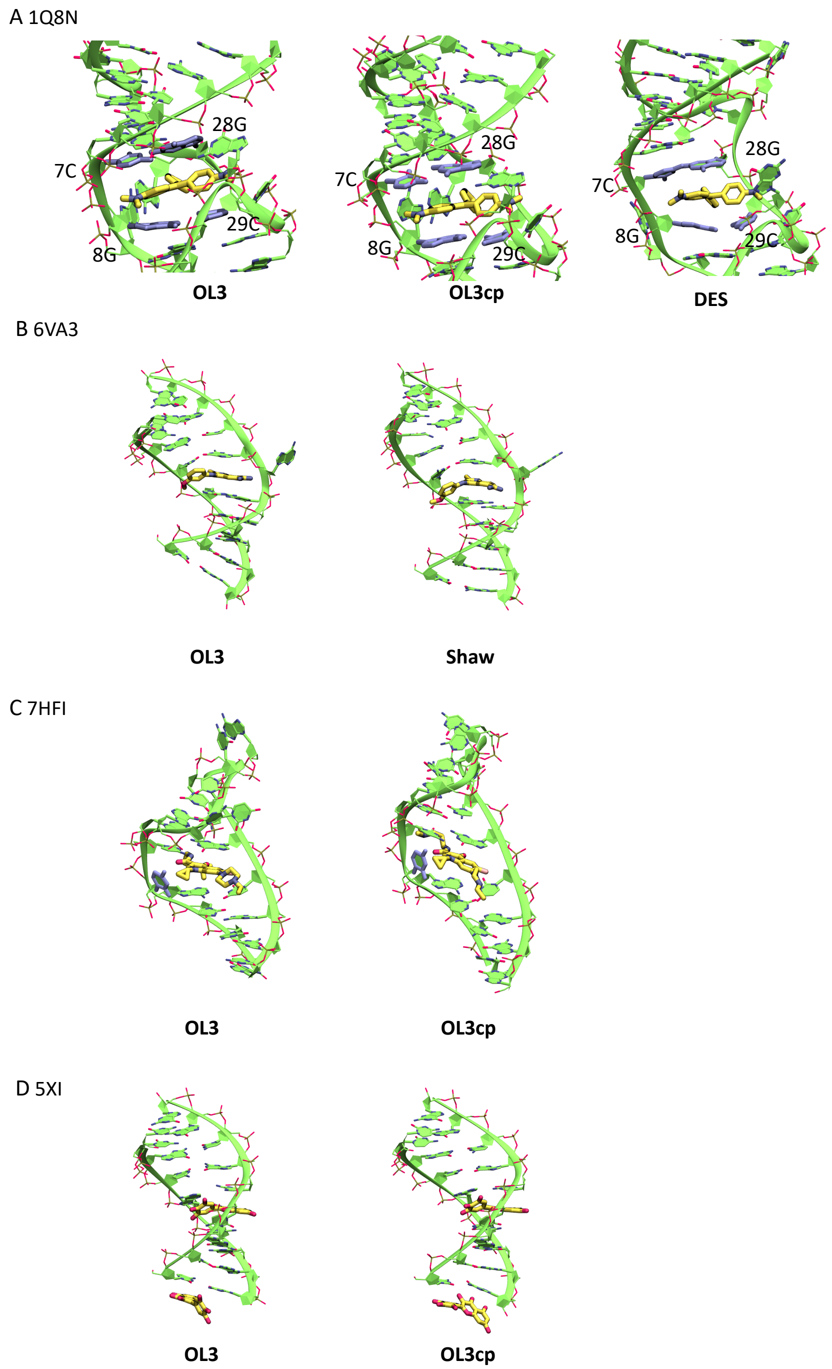

Supplementary Figure S17. RNA structural integrity and ligand positioning across selected force fields. (A) In 1Q8N, OL3 and OL3cp maintain stacking and key base pairs, while DES shows deformation of the A-RNA helix. (B) In 6VA3, OL3 retains the A-form fold, while Shaw promotes partial unfolding and distortion of the bulge region. (C–D) In both 7FHI and 5XI1, OL3 and OL3cp preserve overall RNA geometry and ligand placement without major structural deviations.

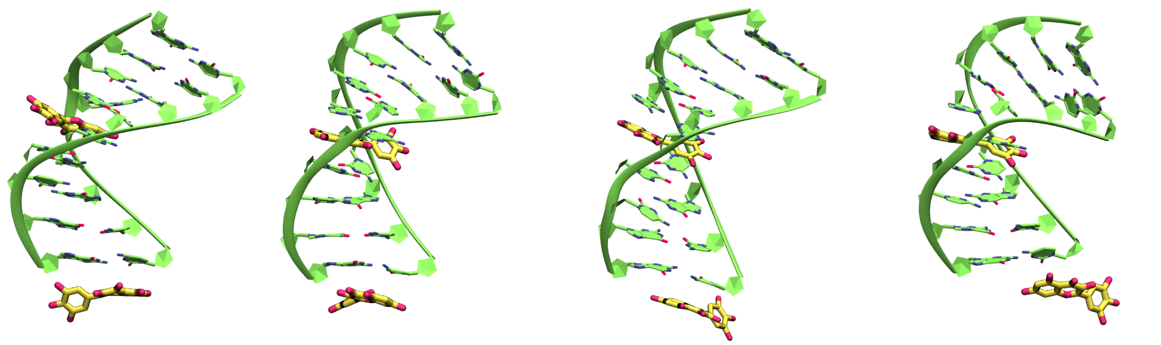

Supplementary Figure S18. Snapshots from Shaw simulations for 5XI1 show the terminal ligand exploring a wide conformational space, indicating weak, transient binding.
